# LncRNA-SNHG5 mediates activation of hepatic stellate cells by regulating NF2 and Hippo pathway

**DOI:** 10.1038/s42003-024-05971-7

**Published:** 2024-03-04

**Authors:** Rongrong Zhang, Yating Zhan, Zhichao Lang, Yifei Li, Weizhi Zhang, Jianjian Zheng

**Affiliations:** Key Laboratory of Clinical Laboratory Diagnosis and Translational Research of Zhejiang Province, The First Affiliated Hospital of Wenzhou Medical University, Wenzhou, 325000 China

**Keywords:** Long non-coding RNAs, miRNAs

## Abstract

Long noncoding RNA small nucleolar RNA host gene 5 (SNHG5) is an oncogene found in various human cancers. However, it is unclear what role SNHG5 plays in activating hepatic stellate cells (HSCs) and liver fibrosis. In this study, SNHG5 was found to be upregulated in activated HSCs in vitro and in primary HSCs isolated from fibrotic liver in vivo, and inhibition of SNHG5 suppressed HSC activation. Notably, Neurofibromin 2 (NF2), the main activator for Hippo signalling, was involved in the effects of SNHG5 on HSC activation. The interaction between SNHG5 and NF2 protein was further confirmed, and preventing the combination of the two could effectively block the effects of SNHG5 inhibition on EMT process and Hippo signaling. Additionally, higher SNHG5 was found in chronic hepatitis B patients and associated with the fibrosis stage. Altogether, we demonstrate that SNHG5 could serve as an activated HSCs regulator via regulating NF2 and Hippo pathway.

## Abstract (web summary)

LncRNA SNHG5 can regulate the Hippo pathway and EMT process by interacting with NF2 to mediate the activation of hepatic stellate cells.

## Introduction

Liver fibrosis, the common process of most chronic liver diseases, will eventually progress to cirrhosis, resulting in high morbidity as well as mortality worldwide^1,2^. Hepatitis virus infection, obesity, alcoholic cirrhosis, and non-alcoholic fatty liver disease (NAFLD) are the major cause of liver fibrosis^3,4^. It has been established that liver fibrosis is caused by the formation of an excessive extracellular matrix (ECM)^5,6^. Liver fibrosis, as a wound-healing response, is often accompanied by crosslinked collagen fibers. During the formation of scars, hepatic stellate cells (HSCs) are activated and play a key role in the production of ECM proteins^7,8^. Currently, the understanding of the HSC activation-related mechanisms remains largely unknown.

The Hippo pathway, a highly evolutionally conserved signaling, has been reported to modulate tissue and organ growth and development, as well as liver size, development, regeneration, metabolism, and homeostasis^9–13^. The Hippo pathway is a serine and threonine-dependent kinase cascade^14^. Once Hippo signaling is switched off, YAP and TAZ, as transcriptional co-activators in the Hippo pathway, depend on the transcriptional enhancer activation domain within the nucleus to exert their regulatory influence on cellular functions^15,16^. In addition, Yap/TAZ are mainly regulated by phosphorylation. It has been found that YAP/TAZ are excluded from the nucleus after being phosphorylated and are generally degraded, resulting in low transcriptional activity mediated by YAP/TAZ^17^. Increasing evidence has demonstrated the importance of the Hippo pathway in HSC activation. Previously, we found that resveratrol inhibits hepatic stellate cell activation via the hippo pathway^18^.

Long noncoding RNAs (lncRNAs), defined as RNA with a length of >200 nucleotides, are incapable of encoding proteins^19,20^. It has been documented that dysregulated lncRNAs play a role in disease progression^21,22^. A growing number of researches have revealed that lncRNA could influence the biological function of RNA, such as mRNA splicing, RNA decay and translation, by binding to DNA/RNA via a complementary sequence^23,24^. Recently, lncRNAs have been reported to serve as HSC activation regulators during liver fibrosis^25–27^.

The small nucleolar RNA host gene 5 (SNHG5), also called U50HG, acts as an oncogene in various human cancers^28,29^. Dysregulation of SNHG5 has been reported in various diseases. For example, upregulation of SNHG5 is found in hepatocellular carcinoma (HCC) and may be associated with HCC prognosis^30^. Over-expression of SNHG5 contributes to the promotion of proliferation, migration and metastasis in cancer cells^31^. Notably, SNHG5 has also been found to be involved in the fibrosis process. Liu et al. found that SNHG5 can inhibit fibrosis of human primary endometrial stromal cells through the Wnt/β-catenin signaling pathway^32^. In this study, we aimed to explore the biological role of SNHG5 in liver fibrosis and its underlying mechanisms.

## Results

### SNHG5 expression is enhanced during liver fibrosis

**Fig. 1:**
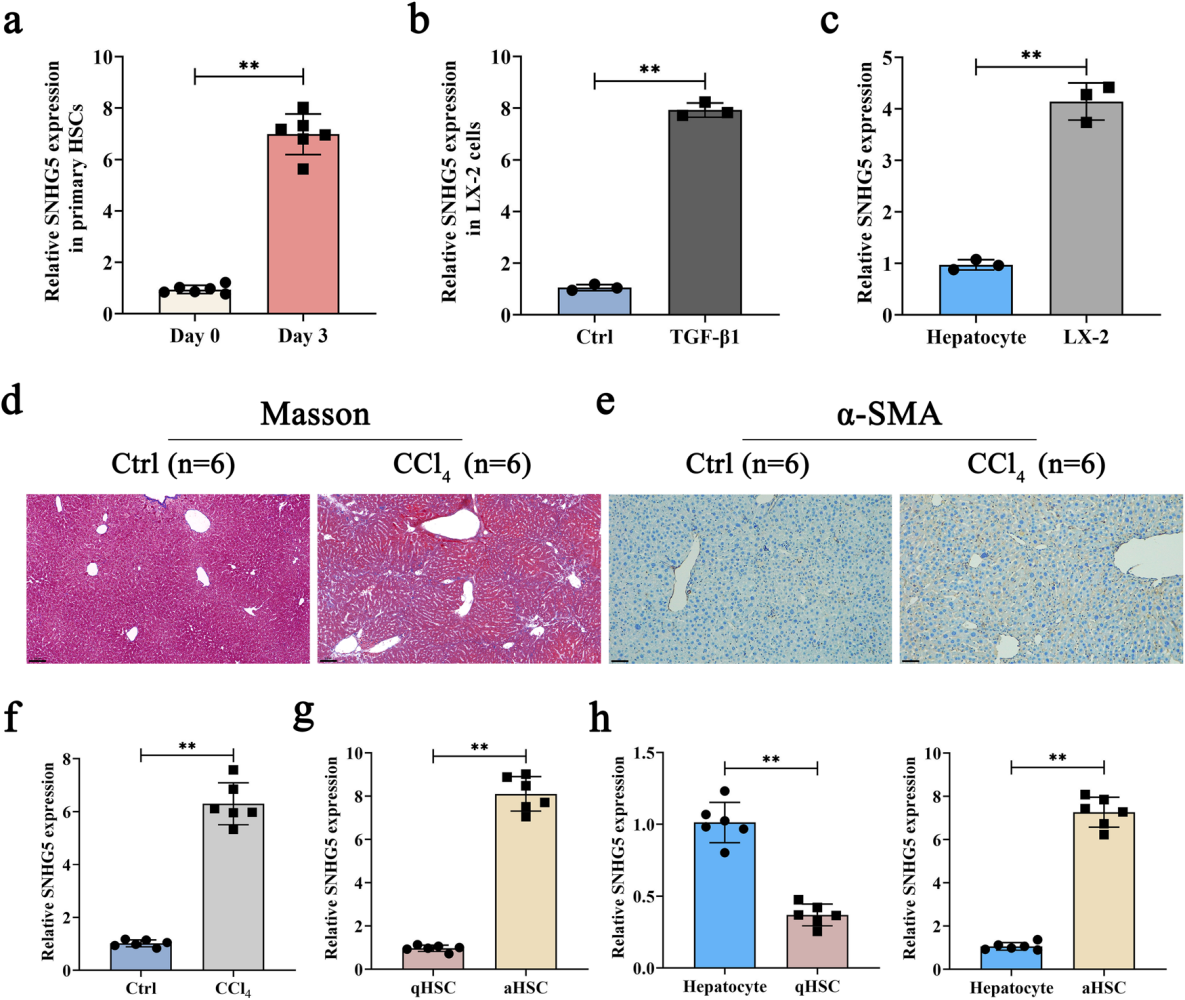
Upregulation of SNHG5 in liver fibrosis. **a** Primary HSCs were isolated from the livers of healthy mice. Primary HSCs were detected at Day 0 and Day 3 for the expression of SNHG5 (*n* = 6 per group). **b** The level of SNHG5 in LX-2 cells treated with TGF-β1 (5 ng × ml^-1^) (*n* = 3 per group). **c** Primary hepatocytes were isolated from healthy mice, cultured for 24 h and harvested for RNA isolation. SNHG5 expression in primary hepatocytes and LX-2 cells (*n* = 3 per group). **d** Masson staining for Collagen and **e** immunohistochemistry staining of α-SMA in CCl_4_-treated mice (*n* = 6 per group). Scale bar, 100 μm. **f** The expression of SNHG5 was examined by qRT-PCR in CCl_4_-treated mice (*n* = 6 per group). **g** Analysis of SNHG5 in primary HSCs isolated from oil- or CCl_4_-treated mice (*n* = 6 per group). **h** SNHG5 was measured in qHSCs (primary 0 day-old HSCs) or aHSCs (primary 3 day-old HSCs) and primary hepatocytes from the livers of healthy mice (*n* = 6 per group). Each value is the mean ± SD of six independent experiments. **P < 0.01.

To determine the possible involvement of SNHG5 in liver fibrosis, the level of SNHG5 was examined in activated HSCs (aHSCs) as well as the fibrotic liver tissues from carbon tetrachloride (CCl_4_)-treated mice. It is known that freshly isolated HSCs, which are considered quiescent phenotypes (qHSCs), will progress into activated phenotypes with culture time^33^. SNHG5 expression was detected in primary HSCs isolated from the livers of healthy mice. Increased SNHG5 and collagen type I alpha 1 chain (Col1A1) (liver fibrosis-related marker) were found in aHSCs compared with qHSCs (Fig. 1a and Supplementary Fig. 1a). Moreover, SNHG5 and Col1A1 levels in transforming growth factor-β1 (TGF-β1)-treated LX-2 cells were higher than that in untreated cells (Fig. 1b and Supplementary Fig. 1b). Interestingly, higher SNHG5 and Col1A1 were found in LX-2 cells when compared with primary hepatocytes (Fig. 1c and Supplementary Fig. 1c). In vivo, analysis of Masson staining showed that CCl_4_ induced a significant increase in collagen accumulation (Fig. 1d). The indication of relevant animal dynamics showed that the longer the administration time of CCl_4_, the higher contents of liver fibrosis markers Col1A1 and α-smooth muscle actin (α-SMA) in mouse liver tissues (Supplementary Fig. 1d). Accordingly, α-SMA immunohistochemical results indicated enhanced α-SMA protein in mice after CCl_4_ treatment (Fig. 1e). Subsequently, it was found that SNHG5 and Col1A1 were upregulated in the livers from CCl_4_-treated mice in comparison with the control (Fig. 1f and Supplementary Fig. 1e). In line with it, upregulation of SNHG5 and Col1A1 was found in primary HSCs isolated from CCl_4_-treated mice in comparison with that in qHSCs from oil-treated mice (Fig. 1g and Supplementary Fig. 1f). Also, SNHG5 was detected in primary HSCs as well as primary hepatocytes isolated from healthy mice. Our results indicated that compared with primary hepatocytes, SNHG5 was down-regulated in primary HSCs and upregulated in aHSCs (Fig. 1h). All these data suggest that SNHG5 may be involved in the progression of liver fibrosis.

### SNHG5 inhibition attenuates liver fibrosis in vivo and in vitro

**Fig. 2:**
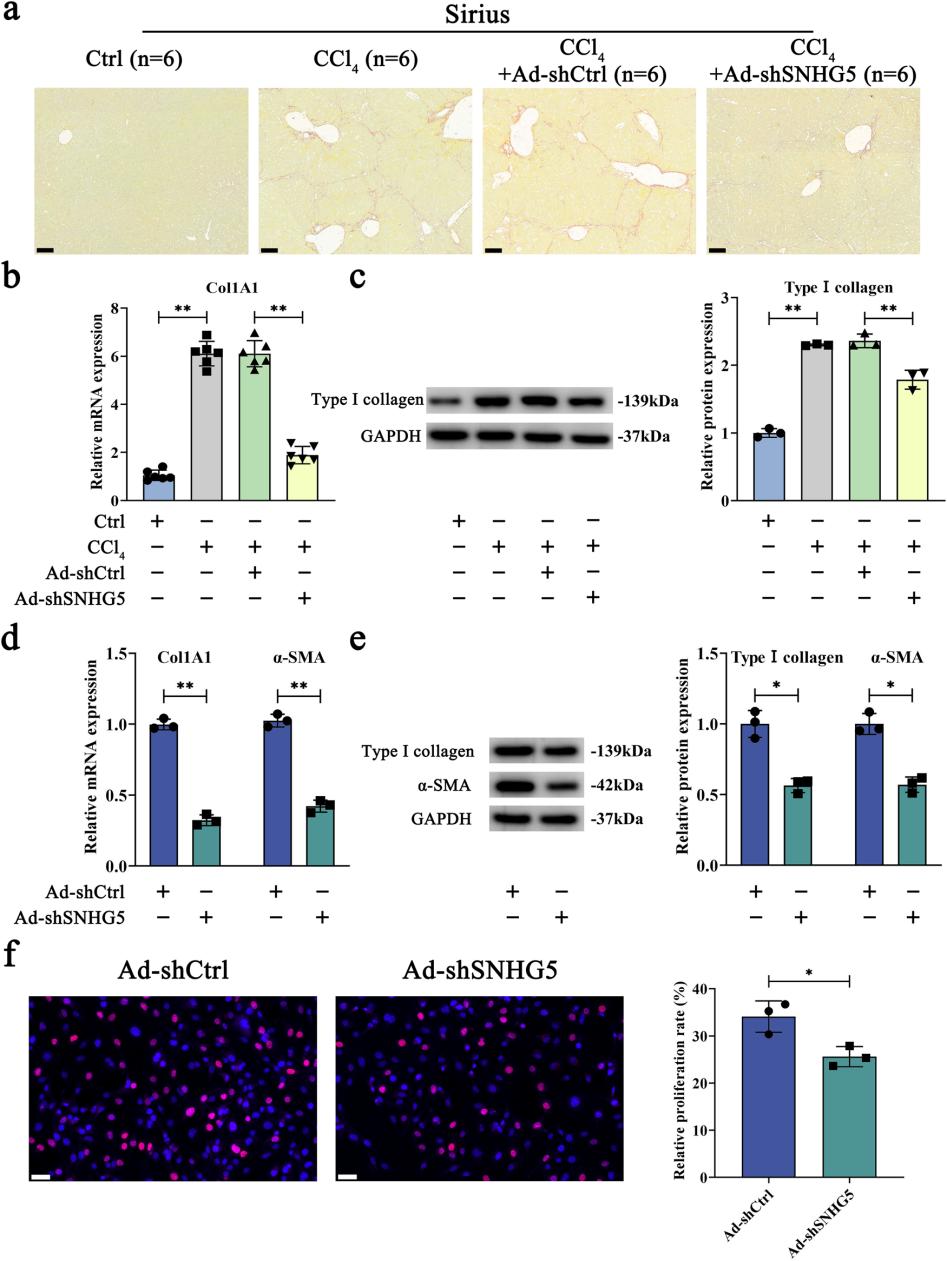
Downregulation of SNHG5 inhibits liver fibrosis in vivo and in vitro. **a** Sirius Red staining was applied to detect collagen deposits (*n* = 6 per group). Scale bar, 100 μm. **b** and **c** Col1A1 expression was inhibited by SNHG5 downregulation in vivo (*n* = 6 per group). The mRNA **d** and **e** protein levels of α-SMA and Col1A1 were determined in primary 0 day-old HSCs following Ad-shSNHG5 treatment for 48 h (*n* = 3 per group). **f** Assessment of HSC proliferation using *EdU* assays in primary HSCs (0 day-old) after treating with Ad-shSNHG5 for 48 h (*n* = 3 per group). Scale bar, 50 μm. Each value is the mean ± SD of three independent experiments. *P < 0.05, **P < 0.01.

To study the functions of SNHG5 in liver fibrosis in vivo and in vitro, adenovirus-mediated shSNHG5-1 (Ad-shSNHG5-1) and Ad-shSNHG5-2 were used to inhibit the expression of SNHG5. Considering the superior efficacy observed upon knockdown, Ad-shSNHG5-1 was selected as the preferred choice for subsequent experimental investigations (Supplementary Fig. 1g). Clearly, Ad-shSNHG5 treatment caused a reduction in SNHG5 expression in the livers of CCl_4_-treated mice (Supplementary Fig. 2a). Reduced SNHG5 was also found in isolated primary HSCs from the fibrotic livers (Supplementary Fig. 2a). As indicated by the results of Sirius Red staining, silencing of SNHG5 contributed to the suppression of collagen accumulation caused by CCl_4_ (Fig. 2a). Similarly, enhanced hydroxyproline caused by CCl_4_ was blocked down by SNHG5 inhibition (Supplementary Fig. 2b). Likewise, CCl_4_-caused type I collagen was attenuated by SNHG5 inhibition (Fig. 2b and Fig. 2c). Further studies demonstrated reduced Col1A1 levels in isolated primary HSCs from CCl_4_-treated mice after Ad-shSNHG5 treatment (Supplementary Fig. 2c). As shown in Supplementary Fig. 2d and Supplementary Fig. 2e, there was no significant change in ALT or AST value between CCl_4_ group and CCl_4_ + Ad-shSNHG5 group. Our data suggest a fibrotic role of SNHG5 in liver fibrosis.

### Silencing of SNHG5 inhibits HSC activation

Activated HSCs are often associated with the enhancement of proliferation, ECM protein and α-SMA synthesis^34^. We next explored the effect of reduced SNHG5 on HSC activation. It was found that Ad-shSNHG5 resulted in the suppression of SNHG5 in vitro (Supplementary Fig. 3a). Ad-shSNHG5 treatment caused an inhibition in the mRNA and protein expressions of α-SMA and Col1A1 (Fig. 2d, e). Results of *5-ethynyl-2’-deoxyuridine (EdU)* assays also showed that Ad-shSNHG5 induced a significant reduction in cell proliferation (Fig. 2f). These data demonstrate that inhibiting SNHG5 contributes to the inactivation of HSC.

### SNHG5 inhibition alleviates HSC activation via Epithelial-mesenchymal transition (EMT)

**Fig. 3:**
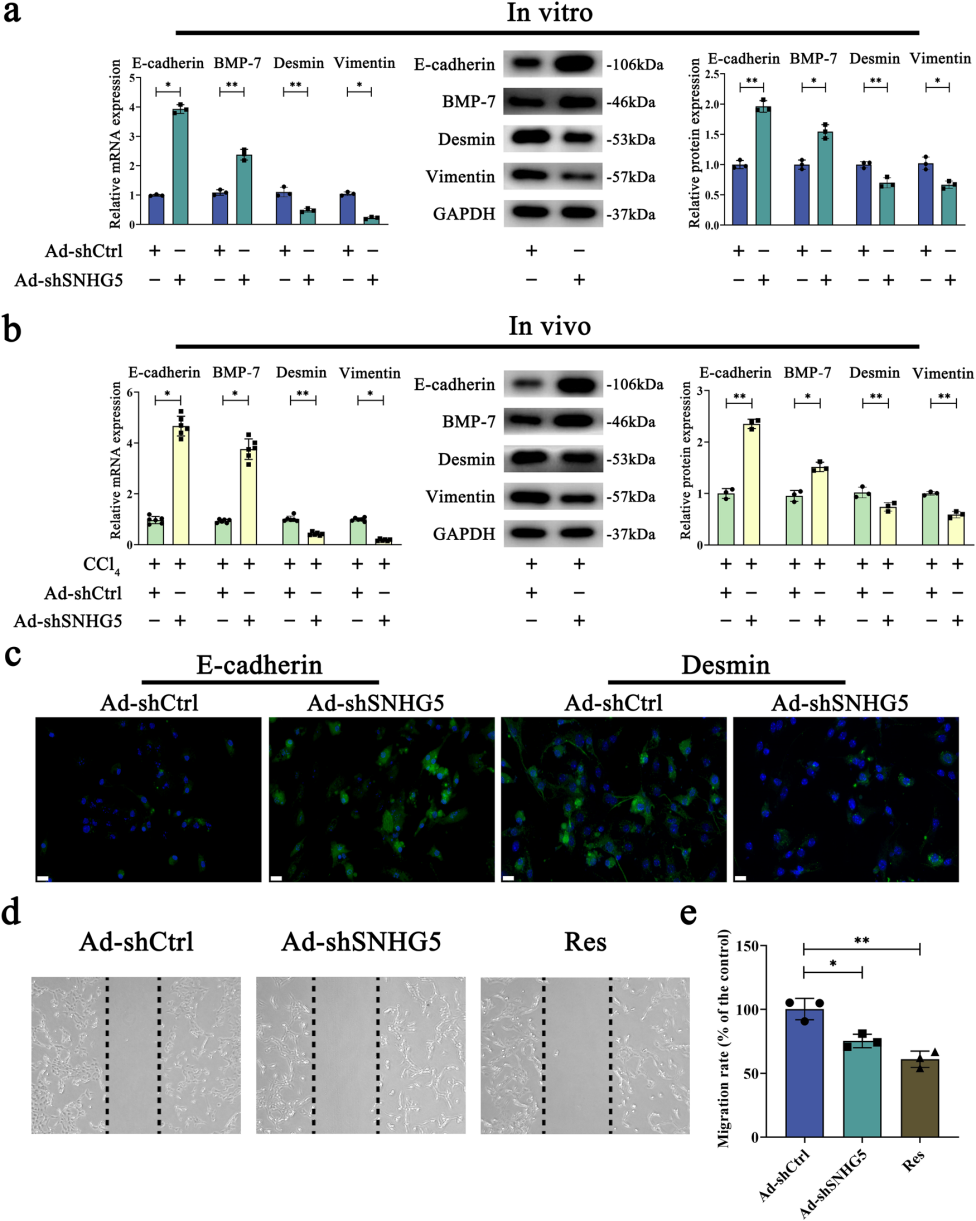
Effects of SNHG5 knockdown on the EMT process. Transduction of primary 0 day-old HSCs with Ad-shSNHG5 was performed for 48 h. The mRNA and protein expressions of E-cadherin, BMP-7, Desmin and Vimentin were measured in SNHG5-knockdown HSCs (in vitro, *n* = 3 per group) (**a**) and in Ad-shSNHG5-treated CCl_4_ mouse livers (in vivo, *n* = 6 per group) (**b**). **c** Desmin and E-cadherin were detected using immunofluorescence staining (*n* = 3 per group). Nuclei were stained with DAPI. Scale bar, 20 μm. **d** A wound healing test was applied to analyze cell migration (*n* = 3 per group). The dashed line shows the edge of cell migration. **e** Measurement of cell migration using Transwell migration assay (*n* = 3 per group). Five fields of migrated cells in the lower side of the transwell were counted with a microscope at × 100. Each value is the mean ± SD of three independent experiments. Resveratrol (Res) was used as a positive control. *P < 0.05, **P < 0.01.

EMT, in which epithelial cells gradually take on a mesenchymal hallmark, has been demonstrated to be linked to HSC activation^35^. Whether the EMT process participates in the effect of SNHG5 on HSC inactivation was subsequently explored. We found that Ad-shSNHG5 led to an increase in E-cadherin and BMP-7 (epithelial marker), and a reduction in Desmin and Vimentin (mesenchymal marker) in primary HSCs (Fig. 3a). The similar results were also found in vivo after Ad-shSNHG5 treatment (Fig. 3b). Consistent with these results, SNHG5 knockdown-inhibited EMT process was found in isolated primary HSCs from CCl_4_-treated mice (Supplementary Fig. 3b). Notably, the knockdown of SNHG5 also inhibited the EMT process in primary hepatocytes derived from CCl_4_-treated mice (Supplementary Fig. 3c). Accordingly, results of immunofluorescence analysis indicated increased E-cadherin as well as reduced Desmin caused by Ad-shSNHG5 treatment (Fig. 3c). The effects of SNHG5 knockdown on cell migration were also explored. Analysis of wound healing and transwell migration assays indicated an inhibitory role of SNHG5 knockdown in HSC migration (Fig. 3d, e). Meanwhile, SNHG5 over-expression had the opposite effect on the EMT process. Delivering adenoviral vectors expressing SNHG5 (Ad-SNHG5) effectively induced an increase in SNHG5 (Supplementary Fig. 4a). Ad-SNHG5 contributed to the EMT process, with increased Desmin and Vimentin as well as reduced E-cadherin and BMP-7 (Supplementary Fig. 4b). Combined with these, we demonstrate that inhibiting SNHG5 leads to the alleviation of HSC activation, at least in part, via suppressing EMT.

### Hippo pathway is involved in the effect of SNHG5 inhibition on HSC activation

**Fig. 4:**
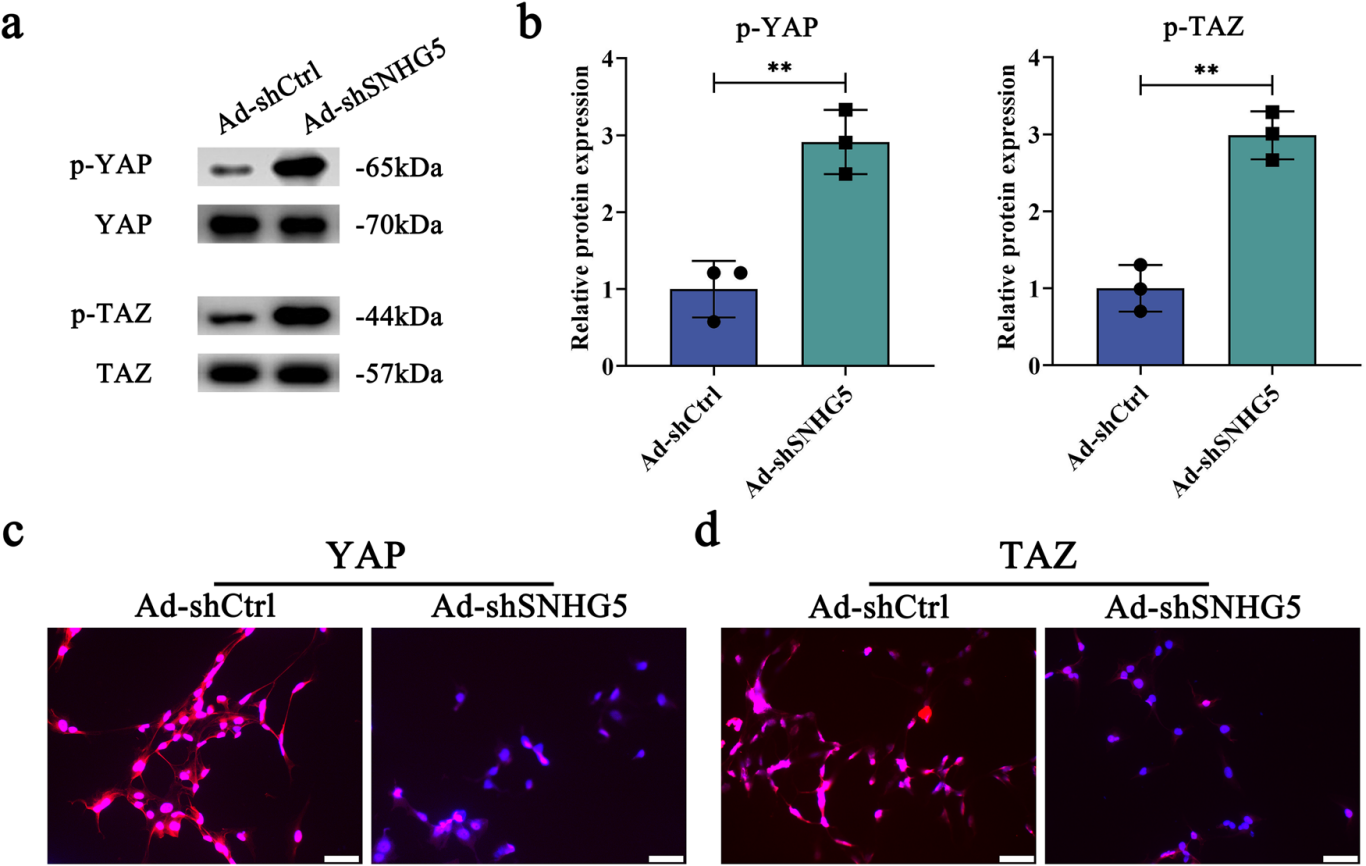
Effects of SNHG5 silencing on the Hippo pathway. Primary 3 day-old HSCs were transduced with Ad-shSNHG5 for 48 h. **a**, **b** The protein expressions of *p*-YAP and *p*-TAZ were increased following the knockdown of SNHG5 in HSCs (*n* = 3 per group). Analysis of YAP (**c**) and TAZ (**d**) protein expression as SNHG5 downregulation by immunofluorescence (*n* = 3 per group). Scale bar, 100 μm. Each value is the mean ± SD of three independent experiments. **P < 0.01.

Recent studies have demonstrated that the Hippo pathway takes a part in the EMT process during HSC activation. Next, the key genes of the Hippo pathway such as YAP and TAZ were examined in cells with SNHG5 knockdown. Results of immunoblot analysis showed that silencing of SNHG5 resulted in the upregulation of p-YAP and p-TAZ (Fig. 4a, b). Immunofluorescence analysis further confirmed the decreased red fluorescence intensity of YAP and TAZ in HSCs after SNHG5 knockdown (Fig. 4c, d). Likewise, overexpression of SNHG5 led to a reduction in p-YAP, p-TAZ, p-MST1 and p-LATS1 (Supplementary Fig. 4c). Taken together, we demonstrate that SNHG5 knockdown inhibits EMT process, at least in part, via activating Hippo pathway.

### SNHG5 interacts with Neurofibromin 2 (NF2)

**Fig. 5:**
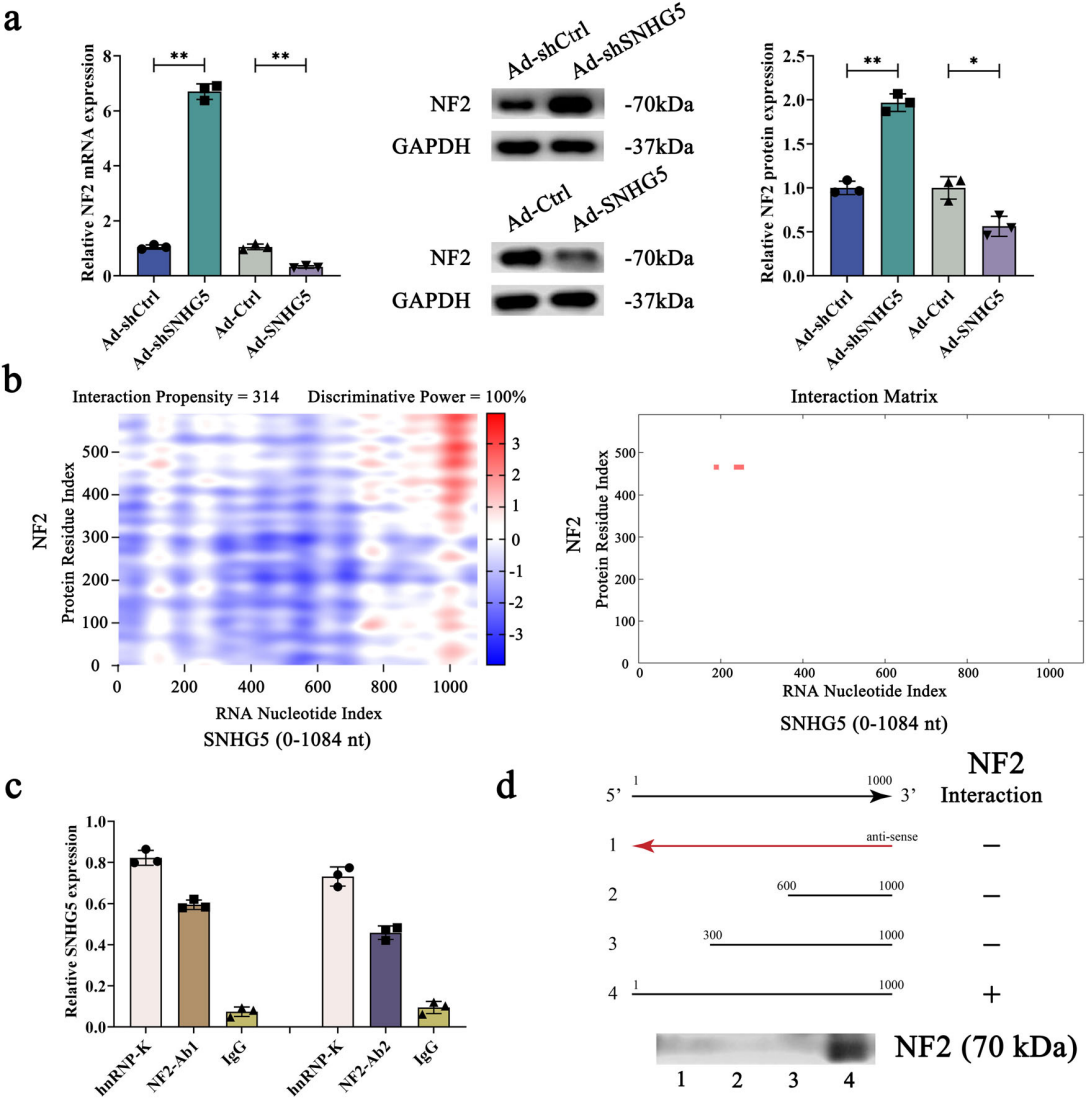
SNHG5 physically interacts with NF2 protein. **a** The protein and mRNA expression of NF2 in primary HSCs with knockdown or overexpression of SNHG5 (*n* = 3 per group). **b** catRAPID was used to predict the overall interaction propensity of SNHG5 and NF2 proteins as well as predict the interaction between SNHG5 (nucleotide position 0–1084 nt) and NF2 proteins (amino acid residues 0–151). **c** RIP experiments were conducted in primary HSC using SNHG5 antibody (*n* = 3 per group). The pull-down NF2 protein was measured by qRT-PCR. Positive and negative controls were hnRNP-K antibody and IgG, respectively. **d** Plot the interactive area between NF2 and SNHG5. Biotinylated RNAs matching different fragments of SNHG5 or their antisense sequences (red lines) were incubated with cell lysates and the related NF2 protein was analyzed by immunoblotting (*n* = 3 per group). Each value is the mean ± SD of three independent experiments. *P < 0.05, **P < 0.01.

Recently, it has been reported that the biological role of lncRNAs could be through regulating RNA-binding proteins. For example, we previously found that lncRNA-MEG3 inhibits HSC activation via SMO protein^35^. Li et al. found that SNHG5 accelerates HCC progression through binding UPF1 and Wnt-signaling pathway^36^. It is known that NF2, also called MERLIN, acts as the main activator for Hippo signaling^37^. Herein, we found that NF2 was upregulated by Ad-shSNHG5 and down-regulated by Ad-SNHG5, respectively (Fig. 5a). Downregulation of NF2 could reverse Ad-shSNHG5-inhibited YAP and TAZ transcriptional activities as well as EMT and HSC activation indicators (Supplementary Fig. 5a). In line with it, SNHG5-mediated EMT process and HSC activation could be reversed by NF2 (Supplementary Fig. 5b). Further studies were performed to explore the relation between SNHG5 and NF2. It has been reported that the potential binding between protein and RNA could be predicted by bioinformatic analysis (catRAPID)^38^. As shown in Fig. 5b, SNHG5 had a possible binding with NF2. This prediction was also confirmed by RPISeq, another bioinformatic analysis. The protein-RNA prediction showed a high propensity of the overall interaction between SNHG5 and NF2 (Fig. 5b). In brief, nt 0–300 of SNHG5 was predicted to interact with NF2. Notably, the interaction between the nt 0–300 position of SNHG5 and NF2 had a discriminative power of 100% (Fig. 5b). Subsequently, nt 0–300 of SNHG5 was selected for the next experiments. The direct binding between SNHG5 and NF2 was examined in HSCs by RNA immunoprecipitation (RIP) using two independent anti-NF2 antibodies. As expected, RIP results indicated the potential binding between SNHG5 and NF2 (Fig. 5c). Analysis of deletion-mapping further confirmed the direct interaction between nt 0–300 of SNHG5 and NF2 protein (Fig. 5d). Unfortunately, no interaction between the nt 300–1000 region of SNHG5 and NF2 protein was found by deletion-mapping analysis. Furthermore, analysis of RIP experiments indicated that compared with the control, reduced SNHG5 enrichment was found in primary HSCs isolated from CCl_4_-treated mice with Ad-shSNHG5 (Supplementary Fig. 5c). Our results demonstrate that the binding of SNHG5 with NF2 protein was inhibited by SNHG5 inhibition in vivo. We mutated the binding sites of SNHG5 (Ad-SNHG5-Mut) to explore the importance of the binding between SNHG5 and NF2 in the Hippo pathway and EMT process. Obviously, SNHG5 down-regulated p-YAP and p-TAZ in the Hippo pathway (Supplementary Fig. 5d and Supplementary Fig. 5e) and enhanced the EMT process (Supplementary Fig. 5f), whereas was inhibited by Ad-SNHG5-Mut. Our results demonstrate that the interaction between SNHG5 and NF2 is crucial for the effect of the SNHG5-mediated Hippo pathway and EMT process.

### SNHG5 expression in chronic hepatitis B (CHB) patients

**Fig. 6:**
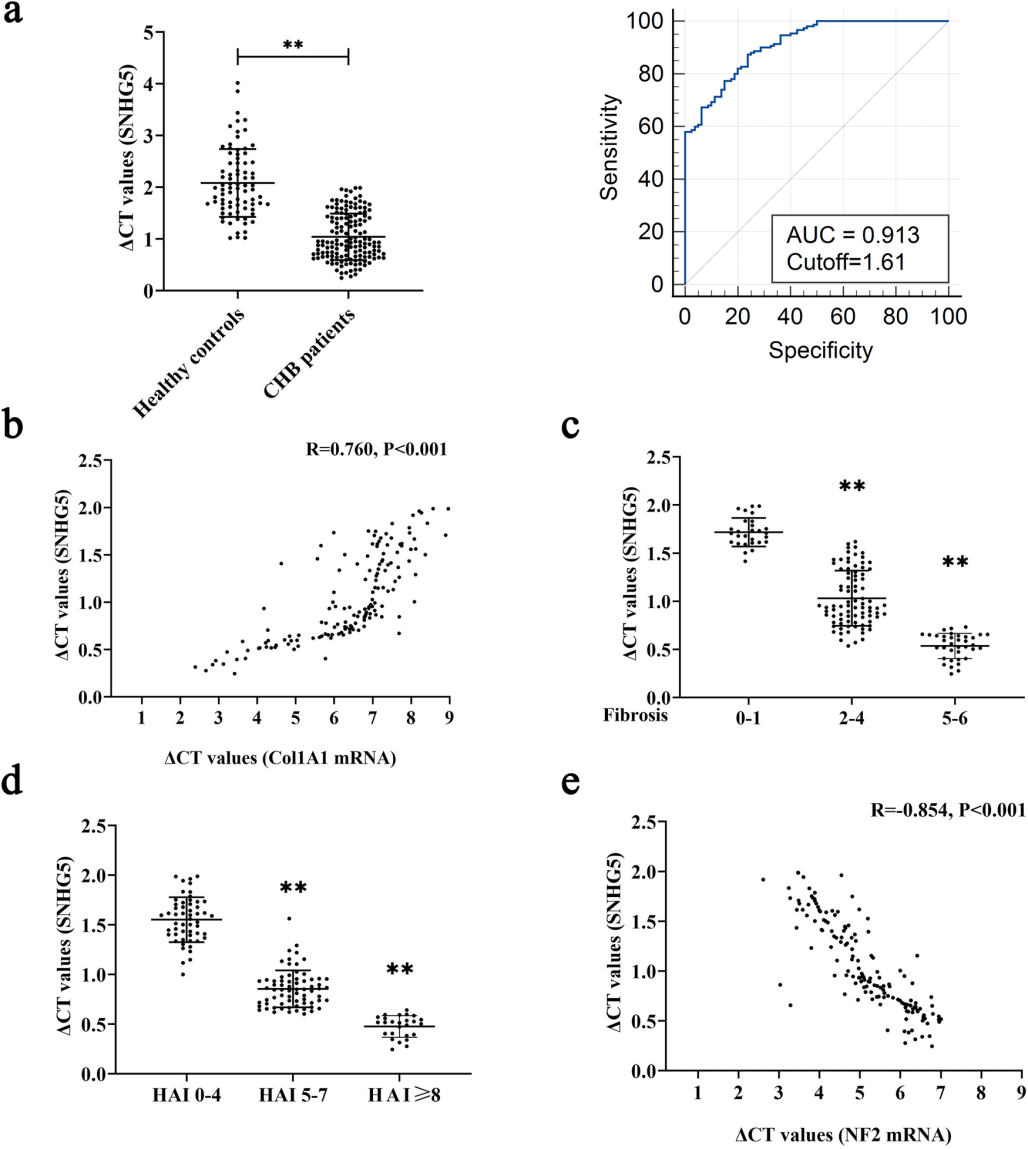
Upregulation of SNHG5 expression in the liver correlates with fibrosis stage in CHB patients. **a** Upregulated level of liver SNHG5 in CHB patients (*n* = 150) in comparison with health Ctrl (*n* = 80) and ROC analysis. **b** Transcriptional level of Col1A1 is positively correlated with SNHG5 in fibrotic human liver tissues (*n* = 150). The statistical analysis was conducted using Pearson’s correlation analysis. **c** ΔCt values of SNHG5 levels in CHB patients with fibrosis score 0–1 (*n* = 28), fibrosis score 2–4 (*n* = 86) and fibrosis score 5–6 (*n* = 36). **d** ΔCt values of SNHG5 levels in CHB patients with HAI score of 0–4 (*n* = 54), HAI score of 5–7 (*n* = 70) and HAI score ≥ 8 (*n* = 26). **e** NF2 expression in fibrotic human liver tissues (*n* = 150). The expression of SNHG5 was calculated via the ΔCt method, normalized to GAPDH, and a smaller ΔCt value suggested higher expression. **P < 0.01.

Finally, we assessed the clinical value of SNHG5 in 150 CHB patients with liver fibrosis as well as 80 healthy controls (Supplementary Table 1). Results of quantitative real-time PCR (qRT-PCR) indicated increased liver SNHG5 in CHB patients in comparison with healthy controls (Fig. 6a). Moreover, ROC curve analysis showed that it yielded an AUC of ROC of 0.913 (95% CI: 0.868–0.946) with 87.3% sensitivity and 76.2% specificity in discriminating CHB patients from healthy controls, when cutoff value is 1.61. Correlation analysis indicated a positive correlation between SNHG5 and Col1A1 (Fig. 6b). We also explored the relationship between liver SNHG5 and fibrosis stage. Interestingly, liver SNHG5 gradually increased with the increased fibrosis scores (Fig. 6c). Similarly, liver SNHG5 was positively correlated with histological activity index (HAI) scores (Fig. 6d). Furthermore, correlation analysis showed a negative correlation between SNHG5 and NF2 (Fig. 6e). In line with it, NF2 expression was down-regulated during HSC activation in vitro *and* in vivo (Supplementary Fig. 6a). In addition, NF2 expression was lower in CHB patients in comparison with healthy controls, and negatively correlated with fibrosis stage as well as HAI score (Supplementary Fig. 6b). All these data suggest that SNHG5 may be a promising biomarker for CHB patients with liver fibrosis.

## Discussion

**Fig. 7:**
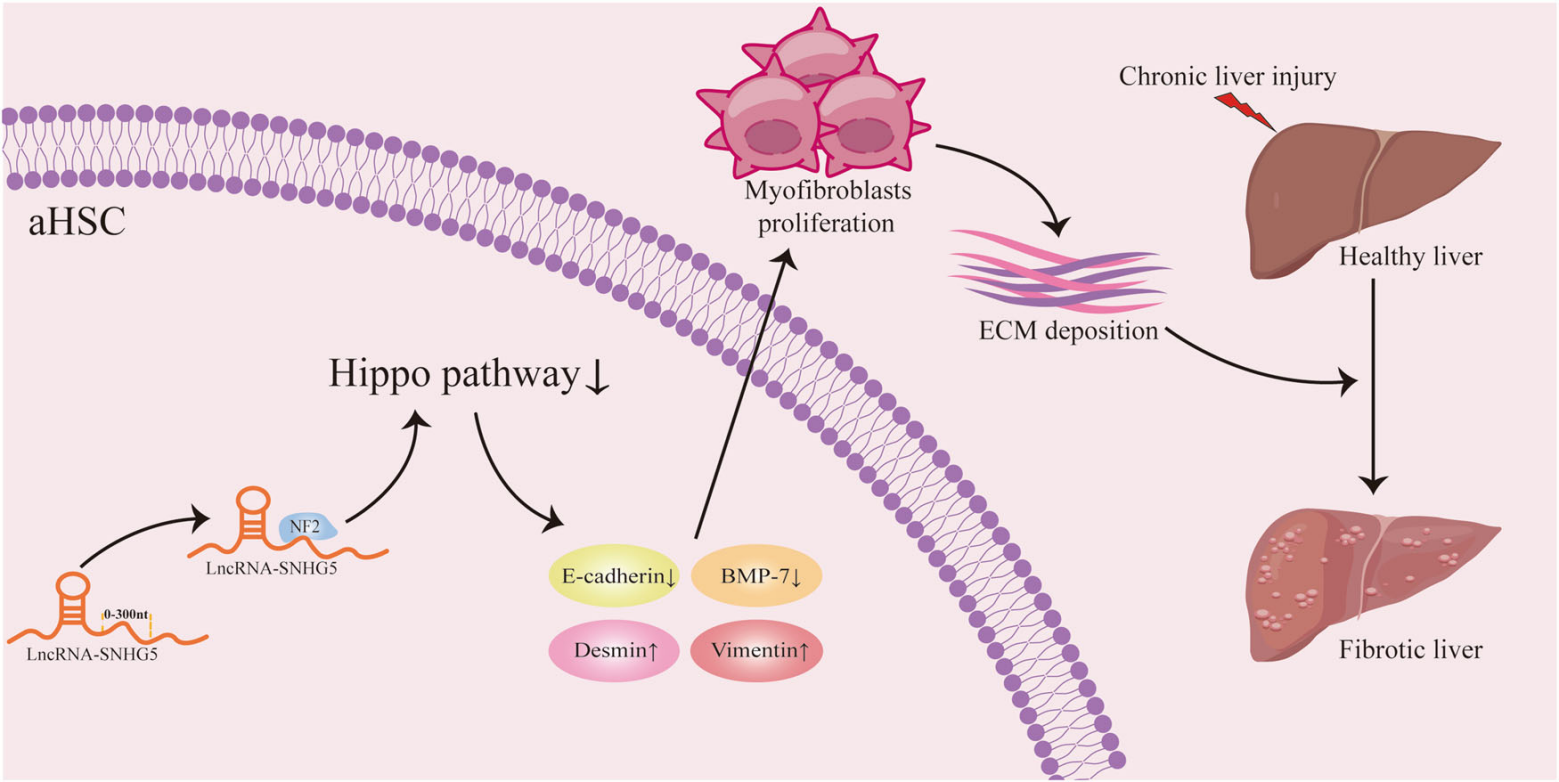
Graphical abstract. Summary of the regulation and mechanism of SNHG5 in liver fibrosis.

Increasing evidence has shown the importance of SNHG5 in cancer progression and metastasis. For instance, Qin et al. found a novel regulatory mechanism of the RBM47/SNHG5/FOXO3 axis on cell proliferation and autophagy in papillary thyroid carcinoma^39^. To date, little is known about the biological role of SNHG5 in liver fibrosis. In this study, we revealed the contribution of SNHG5 in HSC activation as well as liver fibrosis. Owing to the interaction between SNHG5 and NF2, the Hippo pathway was activated by the knockdown of SNHG5, leading to the suppression of the EMT process during HSC activation (Fig. 7).

Increasing evidence has shown the involvement of Hippo pathway in HSC activation. For example, Lee et al. demonstrated that liquiritigenin improves hepatic fibrosis through the Hippo/YAP pathway^40^. Du et al. previously found that YAP-mediated glutaminolysis is important for HSC activation^41^. In addition, 3-tigloyl-khasenegasin F, a natural mexicanolide-type limonoid derivative, was found to suppress HSC activation and liver fibrosis through inhibition of the YAP/Notch3 pathway^42^. We previously also found that Resveratrol inhibits HSC activation, at least in part, via the Hippo pathway-mediated BCL2 and BAX. However, the underlying mechanism for the roles of Hippo/YAP pathway in HSC activation is still largely unknown. Moreover, recent studies have reported that the Hippo pathway-mediated EMT process may be involved in HSC activation^43^. Li et al. found that SNHG5 could induce the EMT process to promote the progression of HCC^30^. Therefore, we determined whether SNHG5 could regulate the EMT process in HSCs via the Hippo pathway in this study. It was found that loss of SNHG5 induced HSC inactivation and EMT process inhibition via the Hippo pathway, with increased p-YAP and p-TAZ. It was also confirmed the contribution of SNHG5 to the negative regulation of the Hippo pathway. Taken together, our data suggest that SNHG5 promotes liver fibrosis, at least in part, through the Hippo pathway-mediated EMT process.

We also explored the mechanism underlying the regulation in the Hippo pathway as a result of SNHG5 inhibition. Bioinformatics analysis revealed a possible interaction between SNHG5 and NF2 (an activator for Hippo signaling), which was further confirmed by RIP experiments. Results of deletion mapping analysis indicated that the position of nt 0–300 of SNHG5 interacted with NF2 protein. Deletion of the NF2 binding site in SNHG5 could effectively inhibit the effects of SNHG5 on the Hippo pathway and the EMT process of HSCs activation. Our results suggest that SNHG5 induces Hippo-mediated EMT processes, at least in part, by interacting with NF2, and that this is an interesting mechanism for regulating liver fibrosis. Combined with these, we demonstrate that SNHG5 has pro-fibrotic potency and that SNHG5 can regulate the Hippo pathway-mediated EMT process in liver fibrosis through binding to NF2.

It has been reported that hepatocytes undergo the EMT process as an important process in the development of liver fibrosis in vivo. When hepatocytes undergo the EMT process, it contributes to the transformation of hepatocytes into fibroblasts, which finally promotes liver fibrosis. For example, Zeisberg et al. suggested hepatocytes-undergoing EMT as a source of MFs, leading to liver fibrosis in vivo^44^. In the present study, we additionally examined whether SNHG5 inhibition affects the EMT process in hepatocytes. In comparison with the control, Ad-shSNHG5 treatment significantly suppressed the EMT process in isolated primary hepatocytes from CCl_4_-treated mice. Therefore, the loss of SNHG5 contributes to the suppression of the EMT process in hepatocytes. In addition, there may be certain limitations in this study. It is known that adenoviral infection may infect multiple liver cell types. In this study, we found that SNHG5 was mainly reduced in primary HSCs and primary hepatocytes in vivo after Ad-shSNHG5 treatment (Supplementary Fig. 7). The inhibitory effects of Ad-shSNHG5 on liver fibrosis are associated with HSC inactivation and hepatocyte EMT inhibition.

Upregulation of SNHG5 level is found in activated HSCs, animal fibrotic liver tissues and CHB patients, similar to the results obtained in cancers^30^. We also evaluated whether liver SNHG5 could serve as a potential biomarker for liver fibrosis in CHB patients. Obviously, SNHG5 expression was positively correlated with HAI scores as well as fibrosis stage and had a good performance in distinguishing CHB patients with liver fibrosis from healthy controls, suggesting that SNHG5 may be a promising marker for liver fibrosis in CHB patients. In addition, the clinical significance of SNHG5 should be validated in larger sample sizes.

In conclusion, we demonstrate that SNHG5 mediates the activation of hepatic stellate cells by regulating NF2 and the Hippo pathway. Our data also suggest that SNHG5 may be a potential biomarker for liver fibrosis in patients with CHB.

## Methods

### Materials

The CCl_4_ and TGF-β1 were purchased from Sigma (St Louis, MO, USA). Antibodies against type I collagen (Affinity, NO. AF7001, 1:1000), α-SMA (Abcam, NO. ab32575, 1:2000), E-cadherin (Sigma, NO. MABT26, 1:1000), BMP-7 (Sigma, NO. MAB4350, 1:1000), Desmin (Proteintech, NO. 16520-1-AP, 1:50000), Vimentin (Proteintech, NO. 60330-1-Ig, 1: 50000), YAP (Proteintech, NO. 13584-1-AP, 1: 5000), TAZ (Proteintech, NO. 23306-1-AP, 1:2000), p-YAP (Affinity, NO. AF3328, 1:1000), p-TAZ (Affinity, NO. AF4315, 1:1000), MST1 (Affinity, NO. DF8430, 1:2000), p-MST1 (Affinity, NO. AF3688, 1:1000), LATS1 (Affinity, NO. AF7669, 1:1000), p-LATS1 (Affinity, NO. AF7169, 1:1000), NF2 (Abcam, NO. ab109244, 1:100000) and GAPDH (Abcam, NO. ab9485, 1:2500) were purchased. Adenoviral vectors expressing the scrambled shRNA (Ad-shCtrl), Ad-shSNHG5, adenoviral vectors expressing a control scrambled sequence (Ad-Ctrl), Ad-SNHG5 and adenoviral vectors expressing NF2 (Ad-NF2) were purchased from Genechem Co.Ltd (Shanghai, China). Animal experimental protocols were approved by the University Animal Care and Use Committee after mice were supplied by the Experimental Animal Center of Wenzhou Medical University.

### Animal experiments

CCl_4_, a medication known to produce toxic liver damage, was used to create a mouse model of hepatic fibrosis. For 6 weeks, 8-week-old male C57BL/6 J mice were injected intraperitoneally twice a week with a 10% olive oil CCl_4_ solution at a dose of 7 μl × g^-1^ per mouse. The injections were replaced with olive oil in the control mice, and the other treatments were the same.

CCl_4_, which was made by combining olive oil and CCl_4_ in a 1:9 ratio, or olive oil at 7 μl × g^-1^, was injected twice a week for 6 weeks to each animal to induce liver fibrosis. Ad-shCtrl or Ad-shSNHG5 (1 × 10^9^ pfu per 100 µl) was injected into the tail vein of CCl_4_-treated mice once every 2 weeks for 6 weeks. 24 mice were divided into four groups: olive oil group (*n* = 6), which served as the control group; CCl_4_ group (*n* = 6); CCl_4_ with Ad-shCtrl group (*n* = 6); and CCl_4_ with Ad-shSNHG5 group (*n* = 6). The liver specimens from the mice were obtained for staining and additional investigation after they were sacrificed under anesthesia.

All experiments involving mice were conducted within the Experimental Animal Center and received approval from Animal Care and Use Committee of Wenzhou Medical University. We have complied with all relevant ethical regulations for animal use.

### Patients and tissues

Liver biopsy tissue samples were taken from 150 CHB patients and 80 healthy controls in the First Affiliated Hospital of Wenzhou Medical University. Written consent was obtained before sample collection. For CHB patients, liver fibrosis was diagnosed by liver biopsy. There were exclusion criteria for the healthy control group. Exclusion criteria for normal subjects: (1) viral infection, such as hepatitis virus, human immunodeficiency virus; (2) alcohol-related liver disease; (3) Obesity-related liver diseases, such as nonalcoholic steatohepatitis and NAFLD; (4) liver cancer; (5) digestive system diseases, cardiovascular system diseases, rheumatic immune system diseases and other systemic disease complications. This study was performed in compliance with the Declaration of Helsinki and approved by the Ethics Committee of the First Affiliated Hospital of Wenzhou Medical University, and informed consent was obtained from all participants. All ethical regulations relevant to human research participants were followed.

### Separation and culture of primary HSCs

HSCs were isolated following previously established protocols^45^. To precisely eradicate Kupffer cells from the liver in C57BL/6 J mice, liposome-encapsulated dichloromethylene diphosphate was given intravenously. Subsequently, hepatic stellate cells were extracted using an improved density gradient centrifugation technique after mouse livers had been perfused in situ. In 35 mm culture dishes filled with complete media made up of DMEM containing 10 percent FBS, the HSCs were inoculated at a density of 4 × 10^5^ per ml. The media was changed every 2 days while the cells were incubated at 37 °C in humid conditions with 5 percent CO_2_. Cells necessary for passaging were confluent after 5–7 days in culture. The survival rate of HSCs was calculated using the Taipan blue staining method.

### Adenoviral transduction

Primary HSCs were transduced with Ad-shSNHG5 or Ad-SNHG5 at multiplicity of infection (*MOI*) of 10, 20, 40, 60, 80 and 100. Transfection efficiency indicated an optimal *MOI* of 80 after 48 h. In the following experiments, 80 *MOI* was used. Cells were collected for further study after transfection.

### Small interfering RNA transfection (siRNA)

The siRNA against NF2 was purchased from Ribo Life Science Co., Ltd. The primers for si-NF2-1, si-NF2-2 and siCtrl can be found in Supplementary Table 2. Cells in each group were seeded in 6-well plates, and transfection was performed strictly in accordance with the Lipofectamine RNAiMAX transfection reagent operating manual.

### Hydroxyproline assay

Hydroxyproline levels in liver were determined using the Hydroxyproline Assay Kit (BioVision, San Francisco, CA). Briefly, liver tissues (50 mg) were homogenized in hydrochloric acid and allowed to hydrolyze at 120 °C overnight. After hydrolysis, the lysate was centrifuged at 12,000 g for 10 min at 4 °C, allowing the supernatant to evaporate to dryness under vacuum. The amount of hydroxyproline in the liver tissues was assessed using the hydroxyproline colorimetric method. Data were normalized by liver weight.

### Western blot analysis

At pH 7.4, liver tissues and cells were lysed in 50 mM Tris-HCl buffer containing 100 mM 2-mercaptoethanol, 2% w × v^-1^ SDS, and 10% glycerol. The proteins were quantified and separated on SDS-PAGE before being transferred to PVDF and blocked with milk. Antibodies against type I collagen, α-SMA, E-cadherin, Desmin, BMP-7, Vimentin, YAP, TAZ, p-YAP, p-TAZ, NF2 and GAPDH were then added and incubated overnight at 4 °C. After that, secondary antibodies (Rockland goat anti-rabbit IgG, 1:2000) were added and incubated for 1 h at 37 °C. Finally, the protein strips were immunoblotted and evaluated according to the manufacturer’s protocols. GAPDH was regarded as the internal reference.

### qRT-PCR

The Total Cellular RNA Kit (Zomanbio, China) was used to extract total RNA from cells, followed by the ReverTra Ace qPCR RT Kit (Toyobo), which was used to reverse transcribe total RNA to cDNA, according to the manufacturer’s instructions. For RT-PCR, we utilized SYBR Green real-time PCR Master Mix (Toyobo, Osaka, Japan). The relative gene expression was calculated using the 2^−ΔΔCt^ technique. The primers for Col1A1, α-SMA, E-cadherin, Desmin, BMP-7, Vimentin, NF2, GAPDH and mouse SNHG5 were designed as described previously^18,35,46^. The primer sequences used in the analysis can be found in Supplementary Table 2. The internal reference gene for RT-PCR was GAPDH.

### Proliferation assay

Cell proliferation was detected by *EdU* assays. After cell transduction with Ad-shSNHG5 or Ad-shCtrl was finished, cells were stained with *EdU* for 12 h. Cell proliferation rate was measured using the Cell-LightTM *EdU* In Vitro Imaging Assay Kit (Guangzhou RiboBio Co., Ltd., cat# C10310-1), and the experimental protocol was carried out in accordance with the manufacturer’s recommendations.

### Scratch assay

Primary HSCs were cultured on day 0 in monolayers with the Ad-shCtrl group or with the Ad-shSNHG5 group, respectively, for 48 h. Subsequently, trauma was formed in the cell monolayers using the tip portion of a 200 μl pipette, and in order to remove the cell debris produced during the process was washed with the medium. A total of three wounds were made on the dish. 24 h later, the wounds were examined with a contrast microscope at ×100 magnification and contrast images of the three wound sites along the scratch line were acquired. Three identical results were obtained in three separate experiments. The migration index was quantified by the following formula: (24 h scratch distance − 0 h scratch distance) / 0 h scratch distance.

### Immunofluorescence

Cells were cultured for 24 h on a glass cover, subsequently fixed with 4% polyacetal for 15 min at room temperature, then washed with PBS. The adhering cells were permeabilized with 0.5% Triton X-100 and then blocked with 10% goat serum for 1 h. After that, the primary antibody was added and incubated at 4 °C overnight, incidentally, suitably diluting the secondary antibody. After DAPI staining, cells were washed gently with PBS three times and photographed by inverted fluorescence microscopy.

### RIP assays

Following the manufacturer’s instructions, the Magna RIP RNA-Binding Protein Immunoprecipitation Kit (Millipore, MA, USA) was used to perform the RIP experiment utilizing two different NF2 antibodies. HnRNP-k was used as a positive control and IgG as a negative control. Primary HSCs were lysed with RIP lysis buffer, and magnetic beads containing the incubating antibodies were incubated with the lysis solution. As a result of eluting the magnetic beads, qRT-PCR was conducted on the RNA complexes pulled down.

### Statistics and reproducibility

With the use of GraphPad Prism V7.0 and SPSS 23.0 (IBM, SPSS, Chicago, IL, USA), data from at least three independent experiments were expressed as the mean ± SD. The differences between the two groups were evaluated using the Student’s *t*-test, while differences among several groups were evaluated using the one-way ANOVA. Differences were considered statistically significant if two-tailed *P* < 0.05 was met, and *P* < 0.01 was regarded as highly significant.

### Reporting summary

Further information on research design is available in the Nature Portfolio Reporting Summary linked to this article.

### Supplementary information

Supporting information

Description of Additional Supplementary Files

Supplementary Data 1

Reporting Summary

## Data Availability

Supplementary Data 1 contains the source data for the graphs in the main figures. Supplementary Fig. 8 contains the original uncropped blot/gel images of the main figures. The other data supporting the findings of this study are available from the corresponding author upon reasonable request.
