# Supplementary material for: LncRNA-SNHG5 mediates activation of hepatic stellate cells by regulating NF2 and Hippo pathway
Source: Commun Biol. 2024 Mar 4;7:266. doi: 10.1038/s42003-024-05971-7 (PMC10912598; doi:10.1038/s42003-024-05971-7)

**Supporting Information**

**Supplementary Tab.1 Patient Characteristics.**

| Parameter | CHB patients | healthy subjects |
| --- | --- | --- |
| Epidemiology |  |  |
| Gender, m/f (%) | 94/56 (62.7/37.3) | 41/39 (51.2/48.8) |
| Age, years, median (range) | 48.7 (34.6-60.1) | 46.8 (30.9-62.5) |
| Virology |  |  |
| HBe antigen positive, n (%) | 47 (31.3%) |  |
| HBe antigen negative, n (%) | 103 (68.7%) |  |
| ALT |  |  |
| Elevated ALT* | 116 (77.3%) |  |
| Normal ALT | 34 (22.7%) |  |
| Fibrosis stage (Ishak) |  |  |
| F0, n (%) | 17 (11.3%) |  |
| F1, n (%) | 11 (7.3%) |  |
| F2, n (%) | 37 (24.7%) |  |
| F3, n (%) | 14 (9.3%) |  |
| F4, n (%) | 35 (23.3%) |  |
| F5, n (%) | 19 (12.7%) |  |
| F6, n (%) | 17 (11.4%) |  |
| HAI |  |  |
| 2, n (%) | 14 (9.3%) |  |
| 3, n (%) | 28 (18.7%) |  |
| 4, n (%) | 12 (8.0%) |  |
| 5, n (%) | 23 (15.4%) |  |
| 6, n (%) | 20 (13.3%) |  |
| 7, n (%) | 27 (18.0%) |  |
| 8, n (%) | 11 (7.3%) |  |
| 9, n (%) | 9 (6.0%) |  |
| ≥11, n (%) | 6 (4.0%) |  |

* >40 U/L.

**Supplementary Tab.2 Primers used in this study.**

| Primer Name | Sequence (5'-3') |
| --- | --- |
| Mouse |  |
| SNHG5-F | TTGTCTTGTCGGTGAGTGAGTTAC |
| SNHG5-R | CAAGTCTCAGAAGCCTCCAAATCC |
| Col1A1-F | CGATGGATTCCCGTTCGAGT |
| Col1A1-R | GAGGCCTCGGTGGACATTAG |
| α-SMA-F | TCTTCCAGCCATCTTTCATTGGGAT |
| α-SMA-R | CCTGTTTTGGCTCCCTATGTCT |
| E-cadherin-F | CAACGATCCTGACCAGCAGT |
| E-cadherin-R | TGTATTGCTGCTTGGCCTCA |
| BMP-7-F | TCCAAGACGCCAAAGAACCAAGAG |
| BMP-7-R | CCTTCAGGTGCAATGATCCAGTCC |
| Desmin-F | TTGTCAGCGAGGCTACACAG |
| Desmin-R | GGCTGGGTGTGATATCCGAG |
| Vimentin-F | AGACCAGAGATGGACAGGTGA |
| Vimentin-R | CTGGTACTGCACTGTTGCAC |
| NF2-F | CGCCAAGTCCCGAGTGG |
| NF2-R | AACAAGCCAGCCCTCTACTG |
| GAPDH-F | AGGAGAGTGTTTCCTCGTCC |
| GAPDH-R | TGAGGTCAATGAAGGGGTCG |
| U6-F | GAAGATTTAGCATGGCCCCTGC |
| U6-R | CAGTGCAGGGTCCGAGGT |
| si-NF2-1 | CUGAUCAGUUAAAGCAAGATT |
| si-NF2-2 | UCUUGCUUUAACUGAUCAGTT |
| si-Ctrl | GGCGCGCTTTGTAGGATTCGA |
| Human |  |
| SNHG5-F | CGAGTAGCCAGTGAAGATAATG |
| SNHG5-R | CACACAACAGTCAAGTAAACC |
| Col1A1-F | TGGCAAAGAAGGCGGCAAAGG |
| Col1A1-R | AGGAGCACCAGCAGGACCATC |
| NF2-F | ACAGAGCTGCTGCTTGGAGT |
| NF2-R | TGTTTCGGATTTCATTCCAC |
| GAPDH-F | AAATCAAGTGGGGCGATGCT |
| GAPDH-R | GTGCTAAGCAGTTGGTGGTG |

**Supplementary Fig. 1 Expression of HSC activation-related markers *in vitro* and *in vivo*. a** The level of Col1A1 in primary HSCs were detected at Day 0 and Day 3 (n=6 per group). **b** The level of Col1A1 in LX-2 cells treated with TGF-β1 (5 ng/mL) (n=3 per group). **c** Col1A1 expression in primary hepatocytes isolated from healthy mice and LX-2 cells (n=3 per group). **d** Col1A1 and α-SMA at different weeks in CCl_4_ mice (n=6 per group). **e** The expression of Col1A1 in CCl_4_ mice (n=6 per group). **f** Analysis of Col1A1 in primary HSCs isolated from oil- or CCl_4_-treated mice (n=6 per group). **g** Levels of SNHG5, Col1A1, α-SMA, E-cadherin and Desmin in Ad-shSNHG5-1 or Ad-shSNHG5-2-treated cells (n=6 per group). Each value is the mean ± SD of six independent experiments. **P*<0.05, ***P*<0.01.


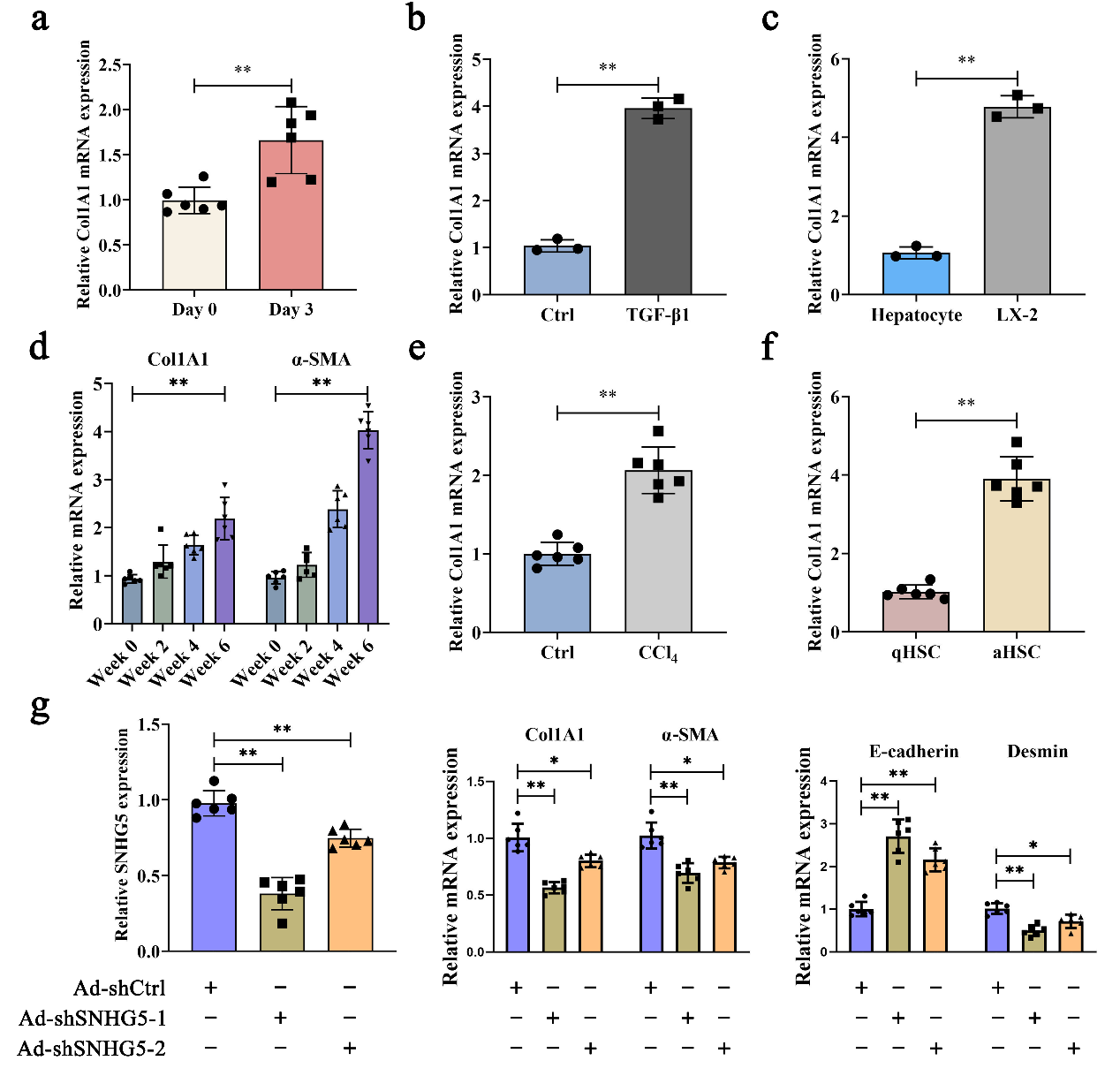


**Supplementary Fig. 2 Effect of loss of SNHG5 on liver fibrosis in CCl_4_-treated mice. a** SNHG5 expression in the liver and isolated primary HSCs (n=6 per group). **b** Hydroxyproline level (n=6 per group). **c** Col1A1 mRNA in isolated primary HSCs (n=6 per group). **d** ALT value (n=6 per group). **e** AST value (n=6 per group). Each value is the mean ± SD of six independent experiments. **P*<0.05, ***P*<0.01.


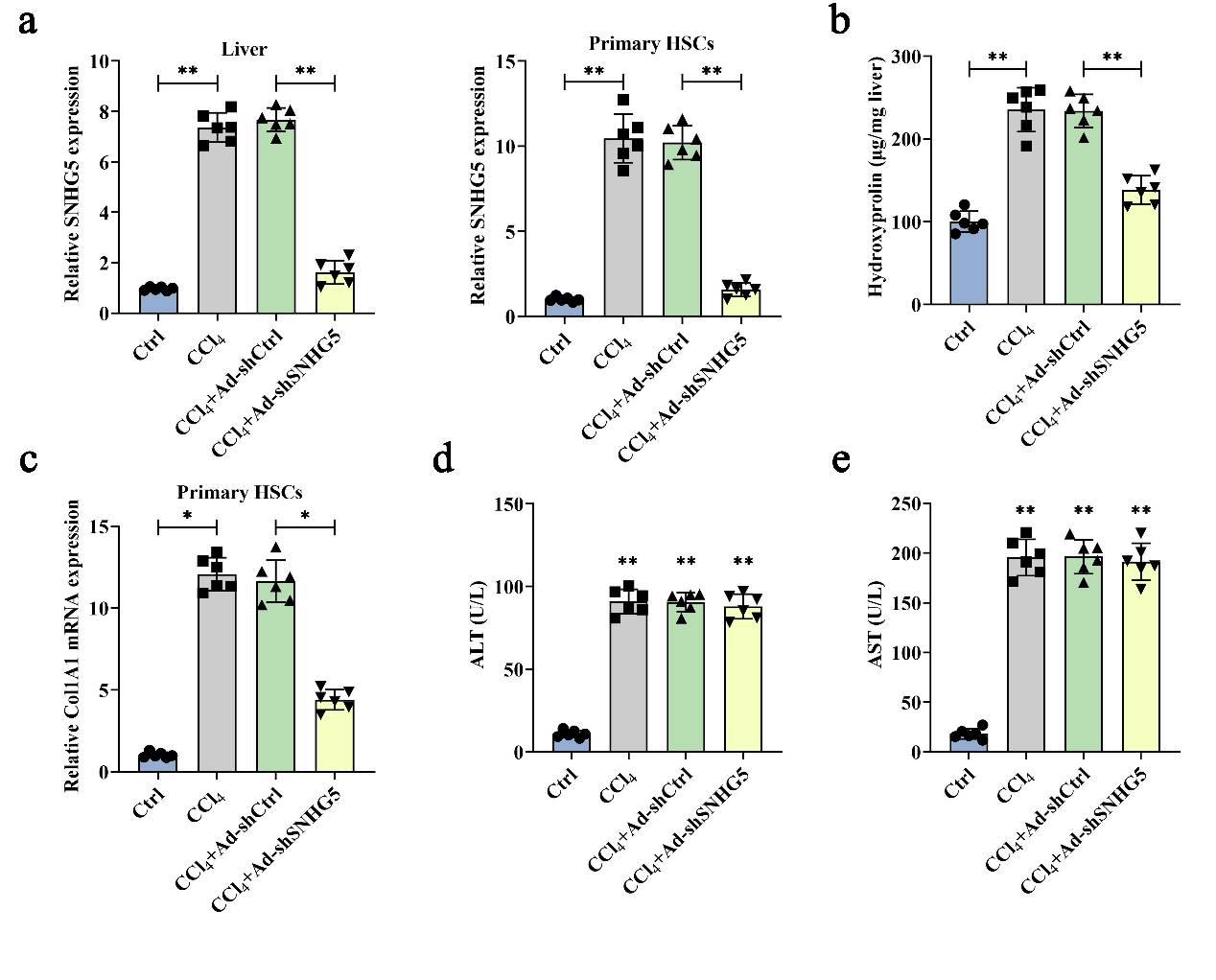


**Supplementary Fig. 3 SNHG5 knockdown inhibits the EMT process.** Primary HSCs and hepatocytes were isolated from CCl_4_ mice after Ad-shSNHG5 treatment. **a** SNHG5 expression (n=3 per group). Primary HSCs at Day 0 were transduced with Ad-shSNHG5 for 48 h. **b** EMT markers in isolated primary HSCs from CCl_4_ mice after Ad-shSNHG5 treatment (n=6 per group). **c** EMT markers in isolated primary hepatocytes from CCl_4_ mice after Ad-shSNHG5 treatment (n=6 per group). Each value is the mean ± SD of six independent experiments. **P*<0.05, ***P*<0.01.


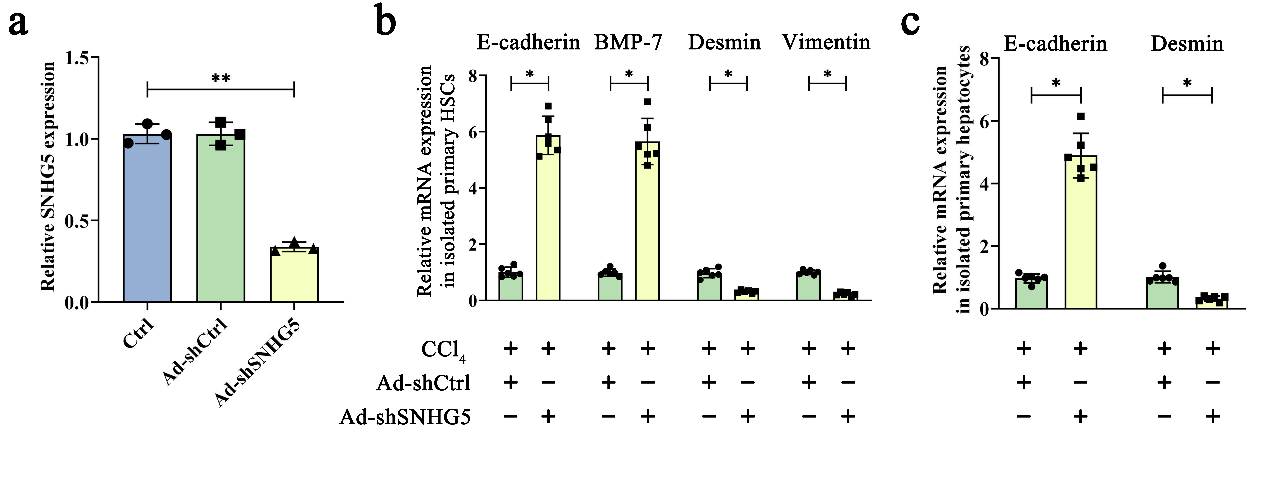


**Supplementary Fig. 4 Effects of SNHG5 upregulation on Hippo and EMT process. a** SNHG5 expression (n=3 per group). **b** EMT markers (n=3 per group). **c** The protein expression of Hippo pathway-related genes (n=3 per group). Primary HSCs at Day 0 were transduced with Ad-SNHG5 for 48 h. Each value is the mean ± SD of three independent experiments. **P*<0.05, ***P*<0.01.


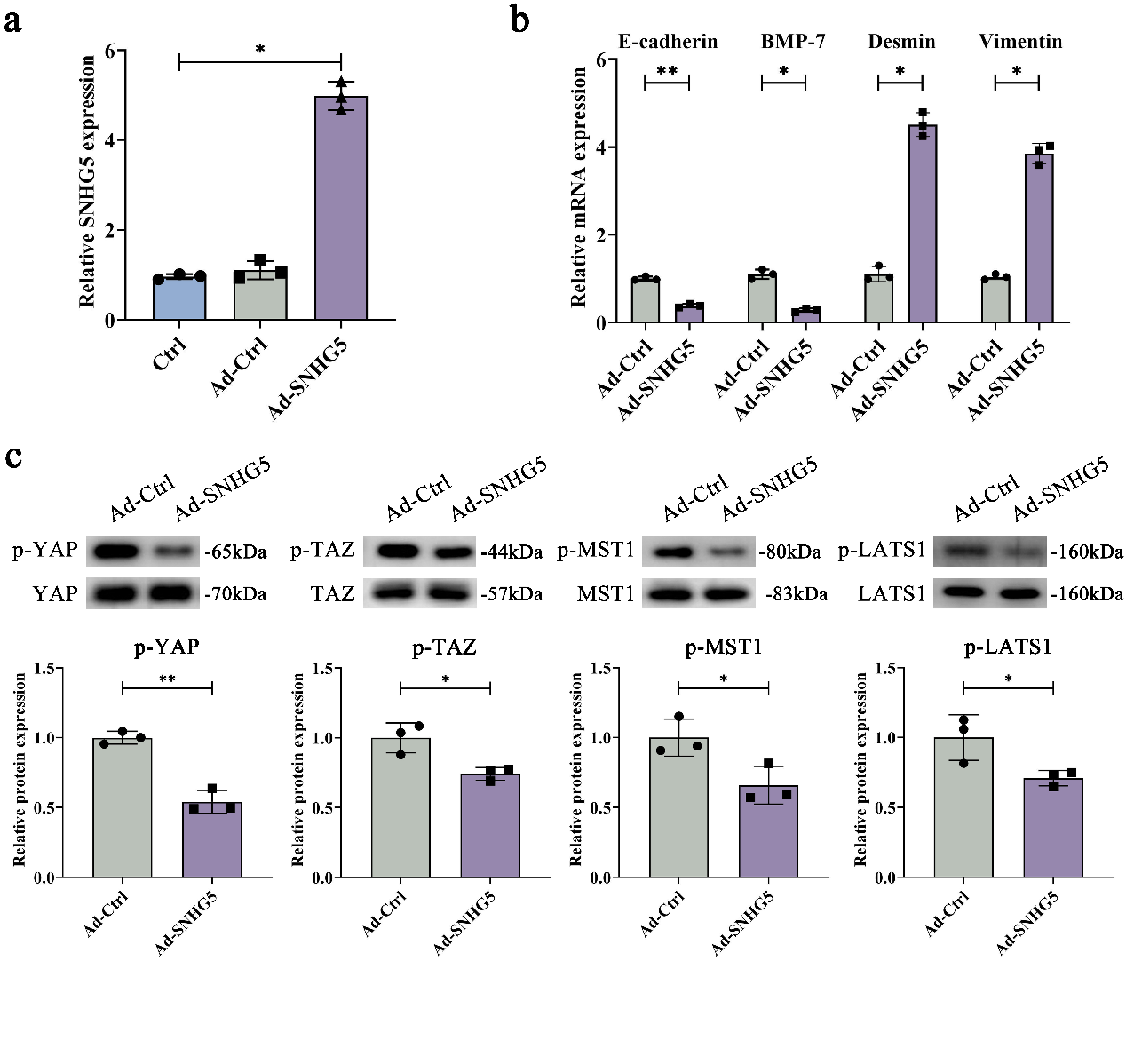


**Supplementary Fig. 5 SNHG5 interacts with NF2 protein. a** The activities of YAP/TAZ, and the levels of Col1A1, α-SMA, E-cadherin and Desmin in cells with SNHG5 and NF2 knockdown (n=3 per group). **b** The levels of Col1A1, α-SMA, E-cadherin and Desmin in cells with SNHG5 and NF2 overexpression (n=3 per group). **c** RIP experiments were performed in primary HSCs using NF2 antibody (n=6 per group). Primary HSCs were isolated from CCl_4_ mice after Ad-shCtrl or Ad-shSNHG5 treatment. qRT-PCR was performed to detect pulled-down SNHG5. hnRNP-K antibody and IgG were used as positive and negative controls, respectively. The core sequence of SNHG5 (nt 0-300 of SNHG5) that binds to NF2 was mutated, which was used as Ad-SNHG5-Mut group. Effects of Ad-SNHG5-Mut on **(d and e)** Hippo pathway (n=3 per group) and **(f)** EMT process in primary HSCs (n=3 per group). Each value is the mean ± SD of three independent experiments. **P*<0.05, ***P*<0.01 compared to Ad-Ctrl group and ^#^*P*<0.05, ^##^*P*<0.01 compared to Ad-SNHG5 group.


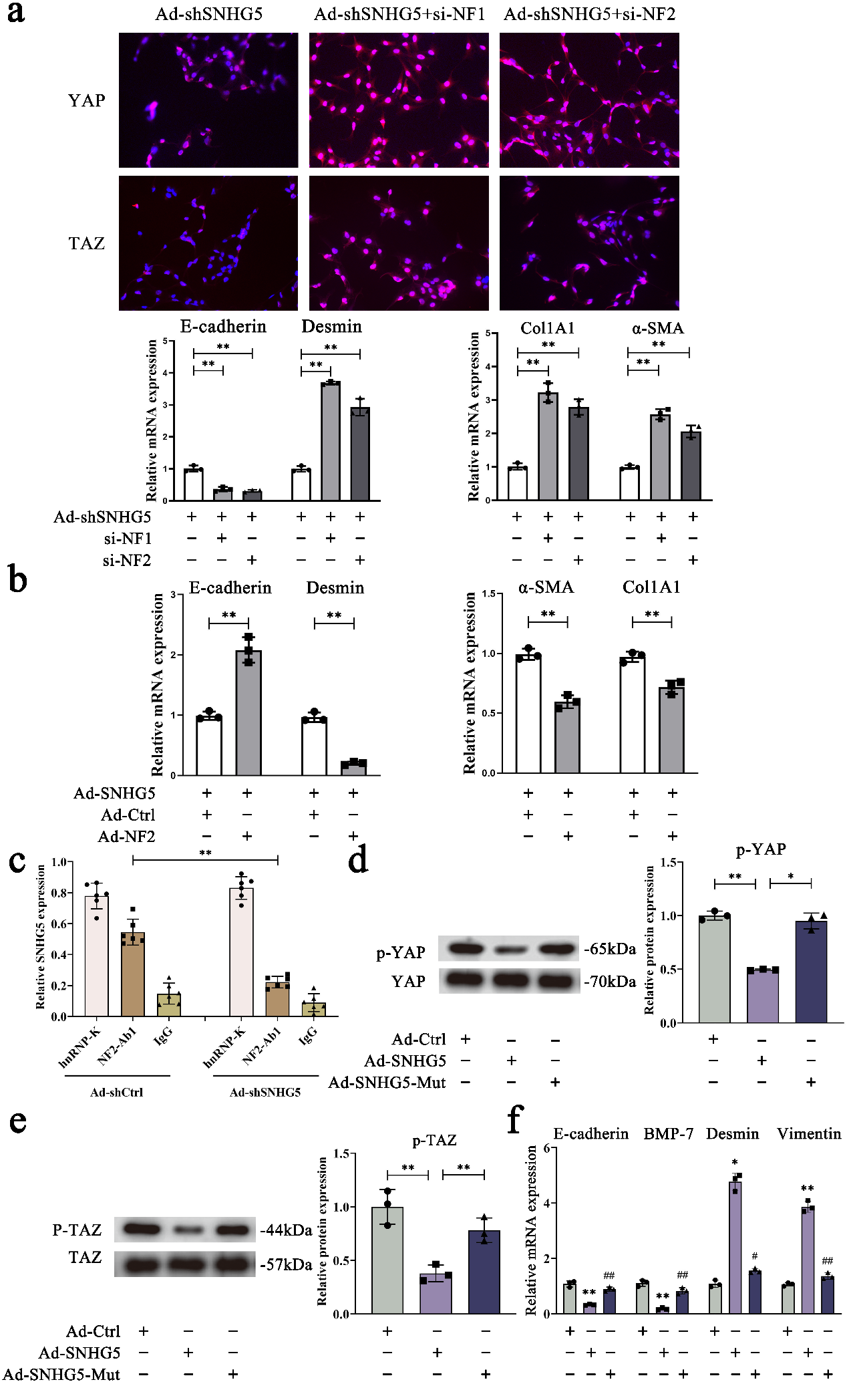


**Supplementary Fig. 6 Expression of NF2 *in vivo and in vitro*. a** NF2 expression was downregulated during HSC activation *in vitro* and *in vivo* (n=6 per group). **b** Down-regulated level of liver NF2 in CHB patients (n=150) in comparison with health Ctrl (n=80). ΔCt values of NF2 levels in CHB patients with different fibrosis scores and HAI scores. **P*<0.05, ***P*<0.01.

**
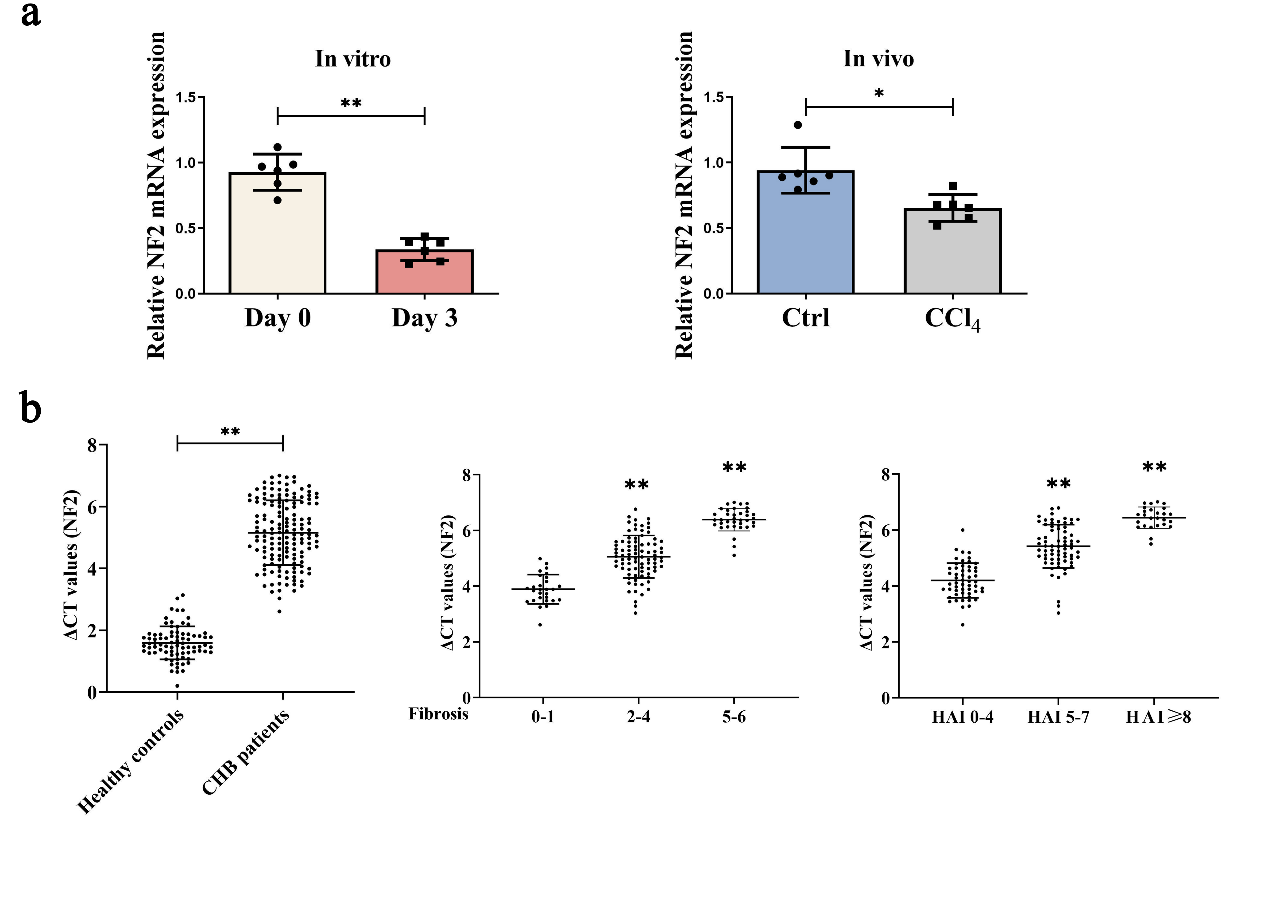
**

**Supplementary Fig. 7 Expression of SNHG5 in various liver cells.** Expression of SNHG5 in liver cells, including hepatocytes, hepatic stellate cells and Kupffer cells (n=6 per group). Each value is the mean ± SD of six independent experiments. ***P*<0.01.


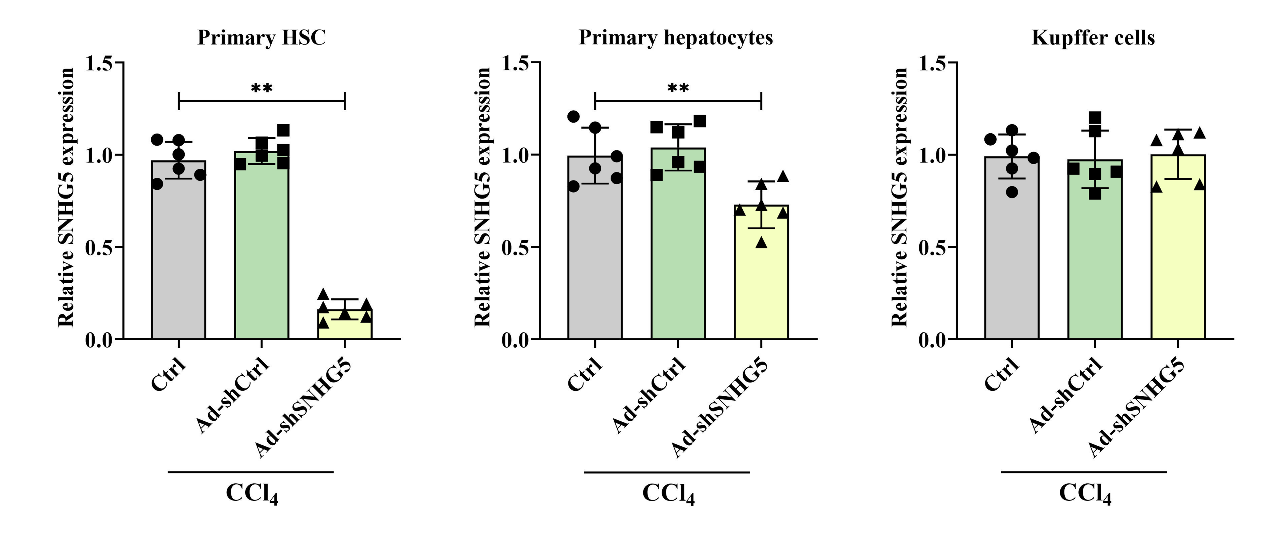


**Supplementary Fig. 8 Unedited blot/gel images.**


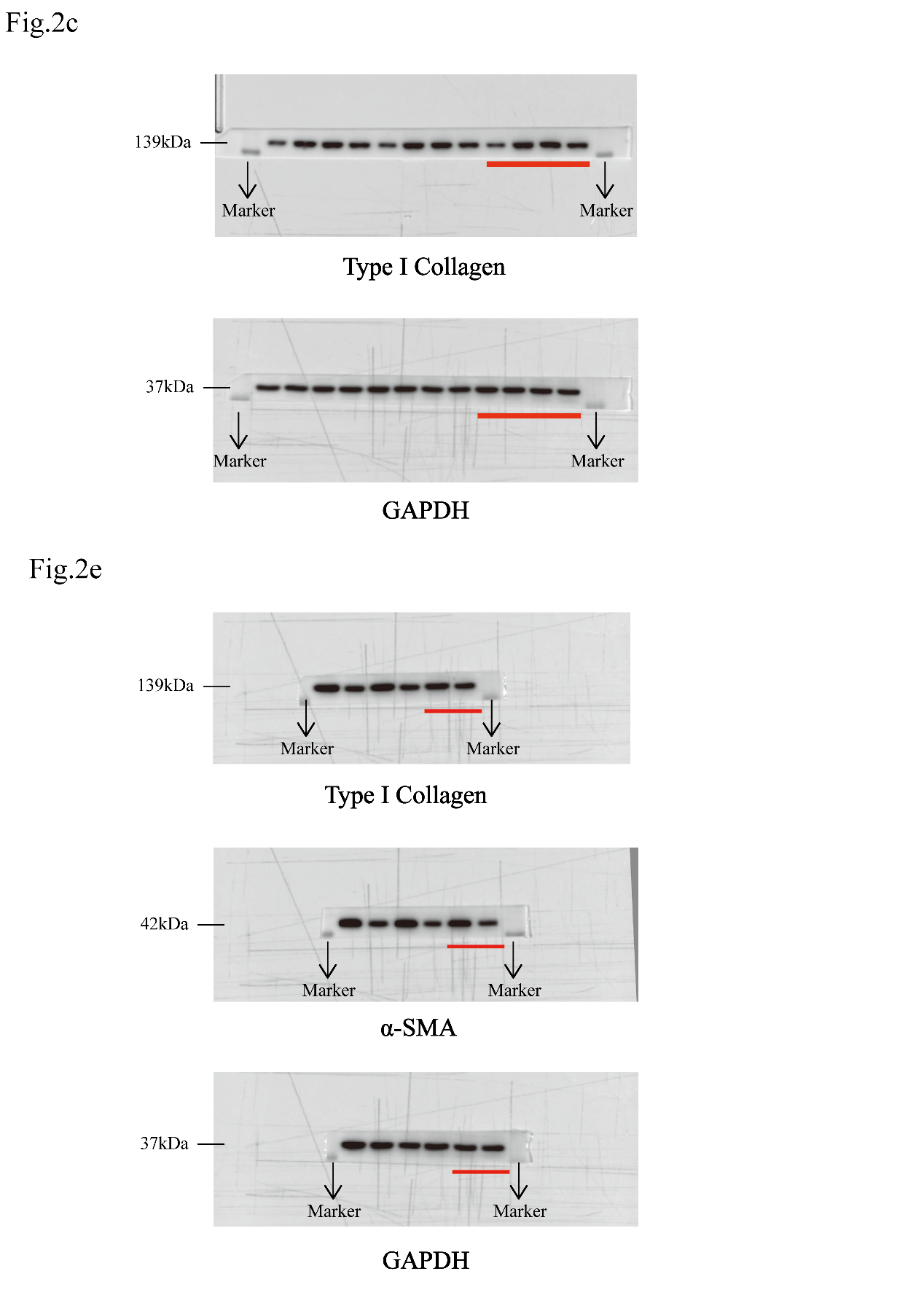


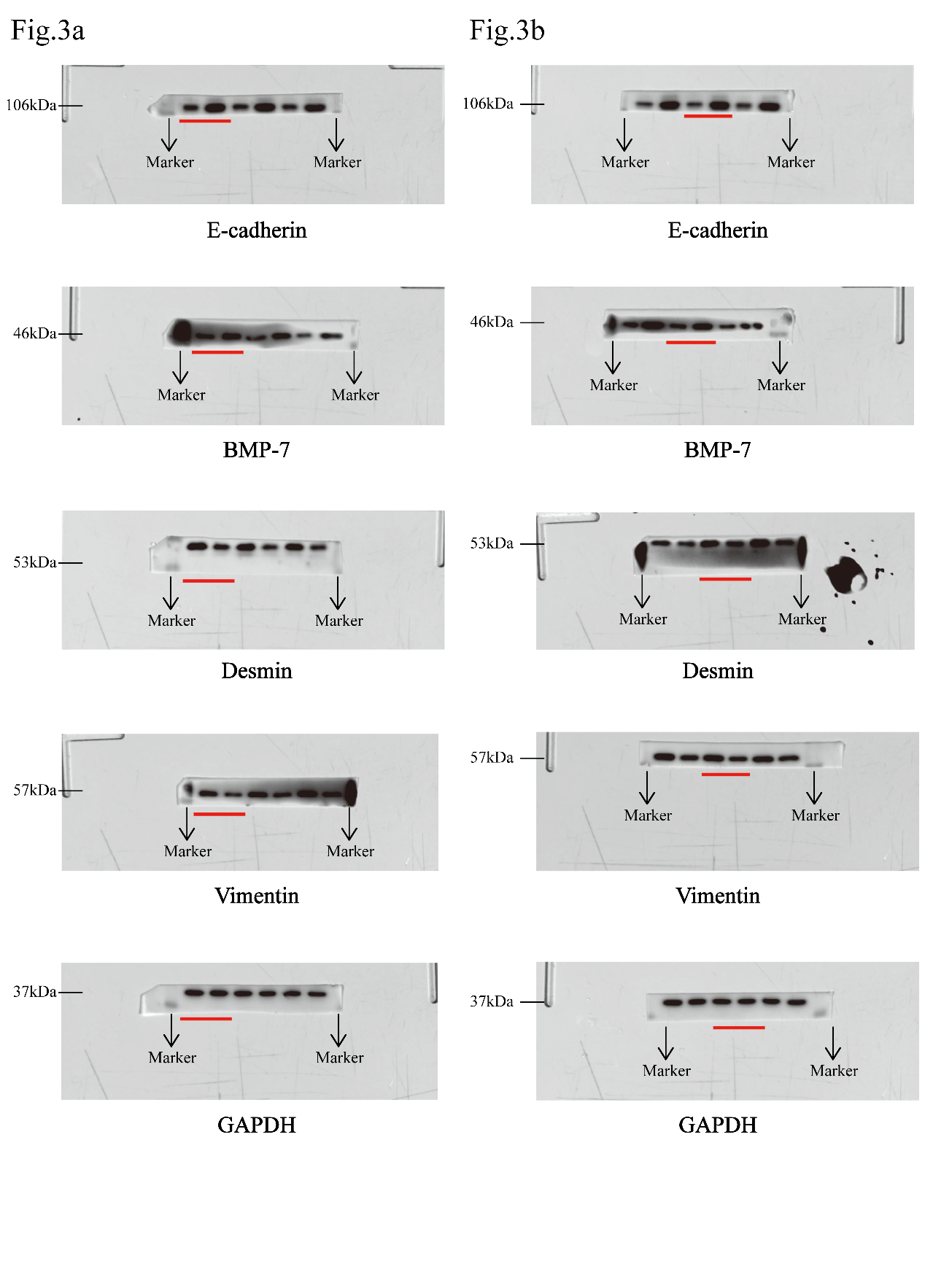


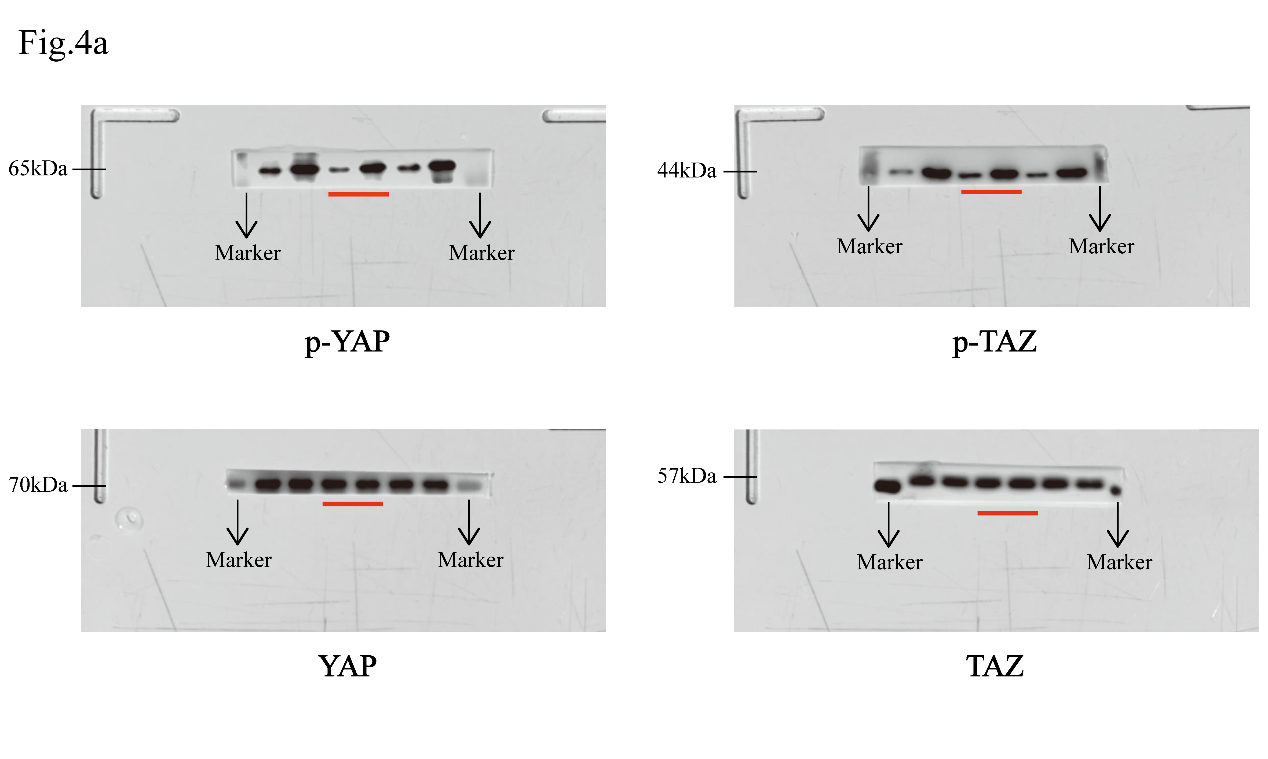


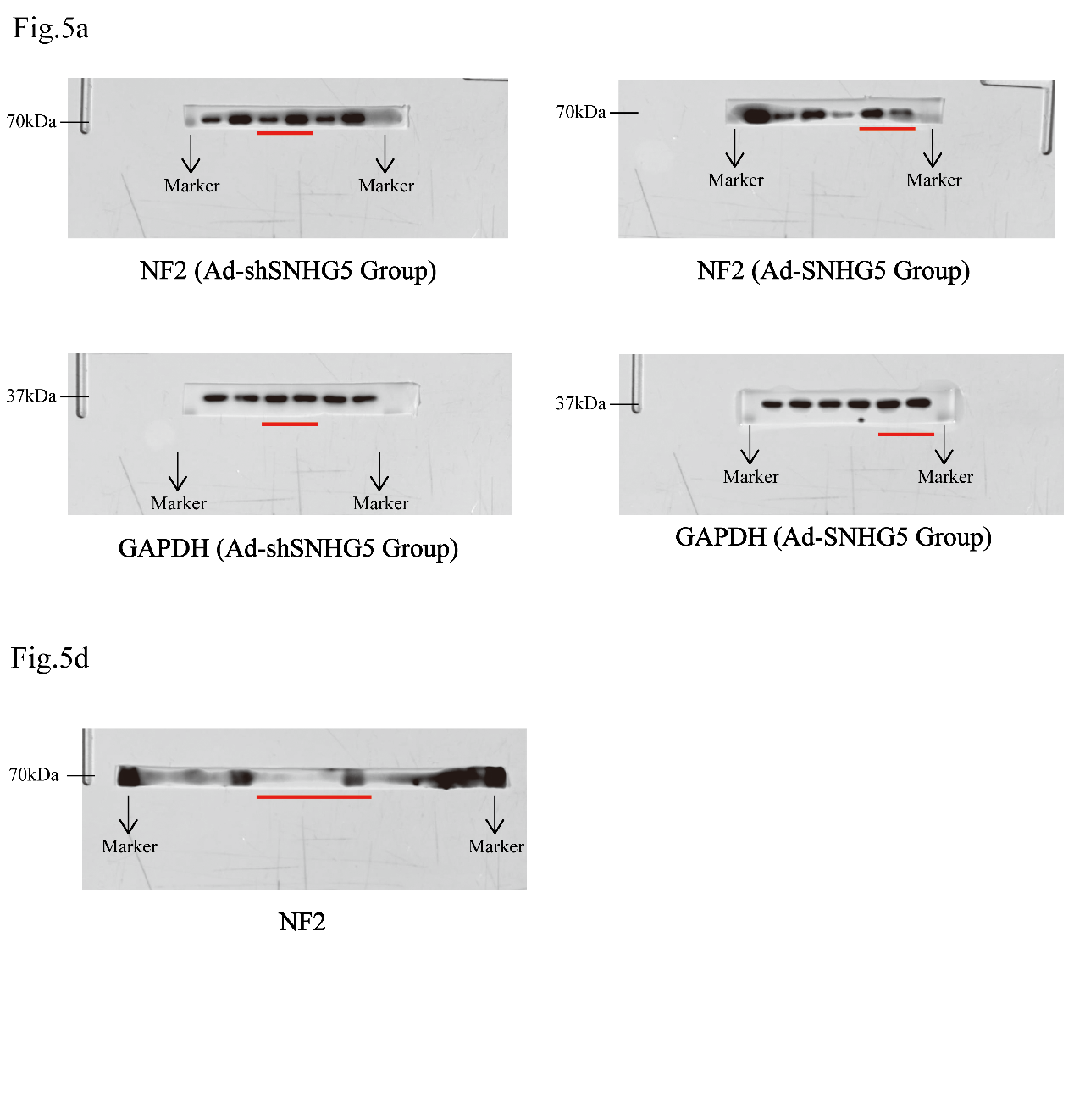


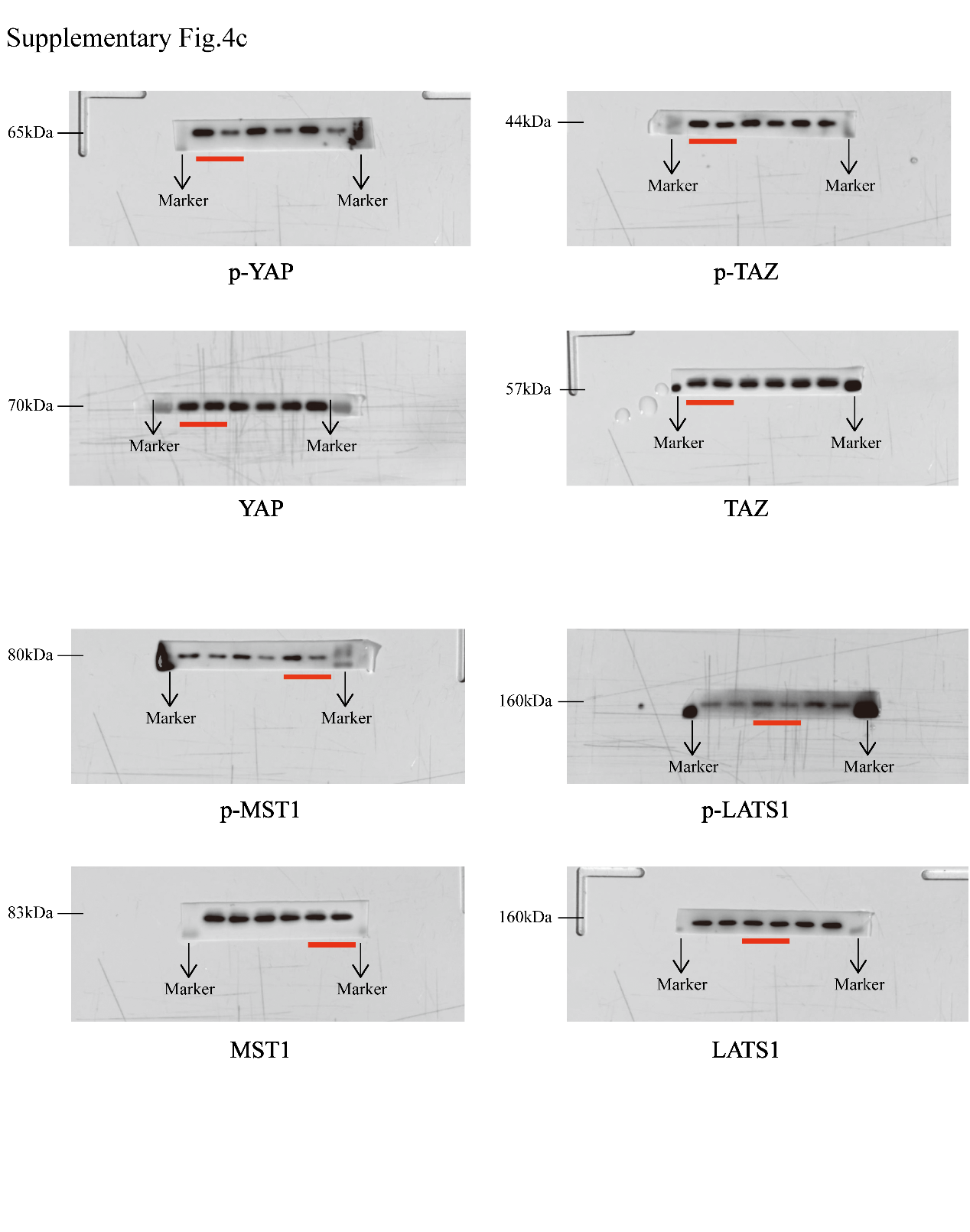


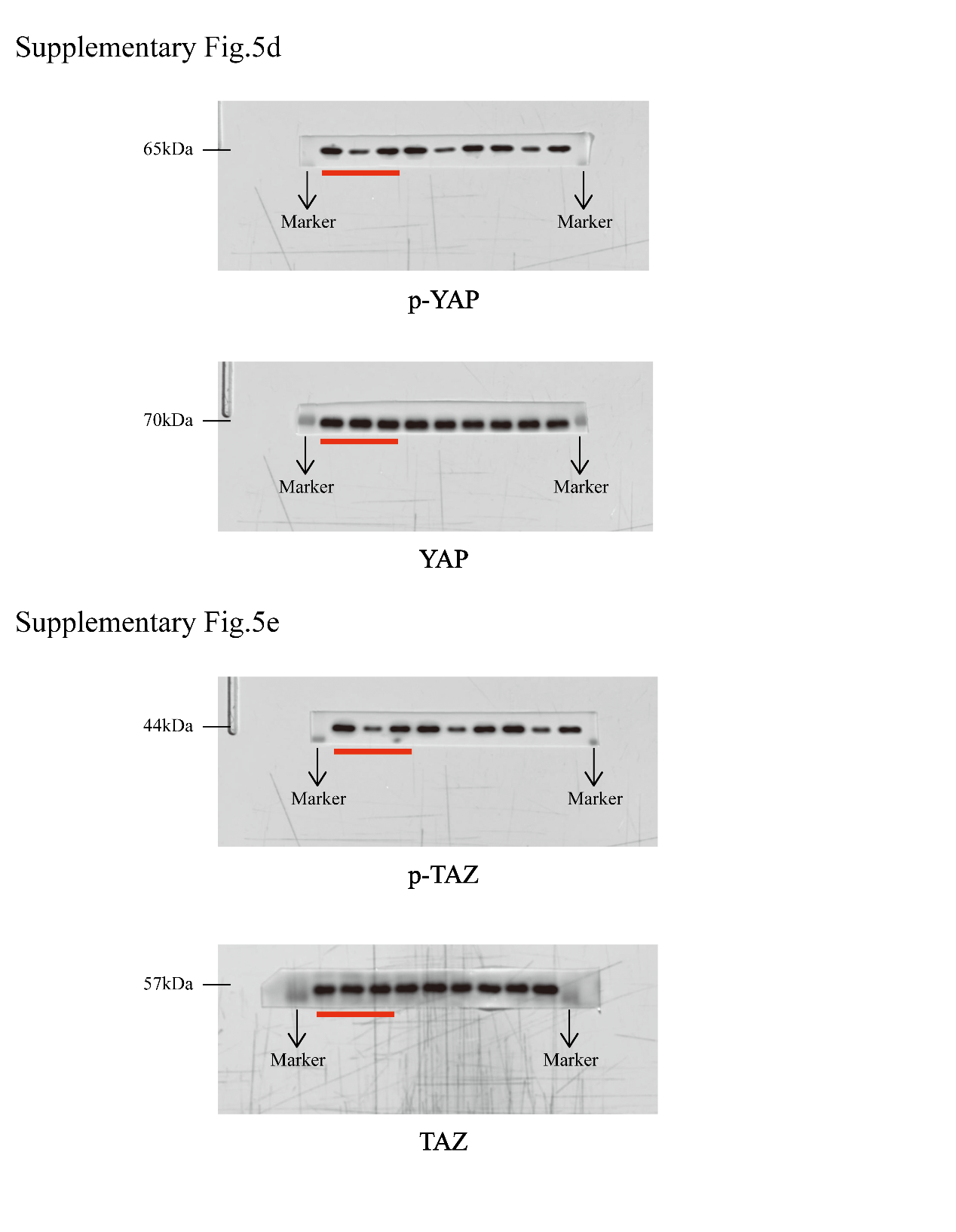

Supplement: Supplementary file 1 — Supporting information [file 42003_2024_5971_MOESM1_ESM.docx]
