# Supplementary material for: LncRNA-SNHG5 mediates activation of hepatic stellate cells by regulating NF2 and Hippo pathway
Source: Commun Biol. 2024 Mar 4;7:266. doi: 10.1038/s42003-024-05971-7 (PMC10912598; doi:10.1038/s42003-024-05971-7)
Supplement: Supplementary file 4 — Reporting Summary [file 42003_2024_5971_MOESM4_ESM.pdf]

nature portfolio

Corresponding author(s): COMMSBIO-23-1316B

Last updated by author(s): Feb 2, 2024

Reporting Summary

Nature Portfolio wishes to improve the reproducibility of the work that we publish. This form provides structure for consistency and transparency in reporting. For further information on Nature Portfolio policies, see our Editorial Policies and the Editorial Policy Checklist.

Statistics

For all statistical analyses, confirm that the following items are present in the figure legend, table legend, main text, or Methods section.

n/a	Confirmed	
☐	☒	The exact sample size (*n*) for each experimental group/condition, given as a discrete number and unit of measurement
☐	☒	A statement on whether measurements were taken from distinct samples or whether the same sample was measured repeatedly
☐	☒	The statistical test(s) used AND whether they are one- or two-sided<br>*Only common tests should be described solely by name; describe more complex techniques in the Methods section.*
☒	☐	A description of all covariates tested
☐	☒	A description of any assumptions or corrections, such as tests of normality and adjustment for multiple comparisons
☐	☒	A full description of the statistical parameters including central tendency (e.g. means) or other basic estimates (e.g. regression coefficient) AND variation (e.g. standard deviation) or associated estimates of uncertainty (e.g. confidence intervals)
☐	☒	For null hypothesis testing, the test statistic (e.g. F, t, r) with confidence intervals, effect sizes, degrees of freedom and P value noted<br>*Give P values as exact values whenever suitable.*
☒	☐	For Bayesian analysis, information on the choice of priors and Markov chain Monte Carlo settings
☒	☐	For hierarchical and complex designs, identification of the appropriate level for tests and full reporting of outcomes
☐	☒	Estimates of effect sizes (e.g. Cohen's *d*, Pearson's *r*), indicating how they were calculated

Our web collection on statistics for biologists contains articles on many of the points above.

Software and code

Policy information about availability of computer code

Data collection	-qRT-PCR was collected by a 7500 rapid quantitative PCR system with SYBR Green master mix.<br>-Fluorescence signals was detected by a confocal laser microscope system (Leica, Wetzlar, Germany).
Data analysis	-Statistical analysis was performed with SPSS 23.0 software and GraphPad Prism V7.0.<br>-Western blotting were quantified by ImageJ software.

For manuscripts utilizing custom algorithms or software that are central to the research but not yet described in published literature, software must be made available to editors and reviewers. We strongly encourage code deposition in a community repository (e.g. GitHub). See the Nature Portfolio guidelines for submitting code & software for further information.

Data

Policy information about availability of data

All manuscripts must include a data availability statement. This statement should provide the following information, where applicable:

- Accession codes, unique identifiers, or web links for publicly available datasets
- A description of any restrictions on data availability
- For clinical datasets or third party data, please ensure that the statement adheres to our policy

The data supporting the findings of this study are available upon request from the corresponding author.

Human research participants

Policy information about studies involving human research participants and Sex and Gender in Research.

Reporting on sex and gender	Sex and gender were not considered in study design.
Population characteristics	See above
Recruitment	150 cirrhotic patients and 80 healthy volunteers were included according to the pathological diagnosis after liver biopsy.
Ethics oversight	The studies involving human participants were reviewed and approved by the Human Research Ethics Committee in the First Affiliated Hospital of Wenzhou Medical University.

Note that full information on the approval of the study protocol must also be provided in the manuscript.

Field-specific reporting

Please select the one below that is the best fit for your research. If you are not sure, read the appropriate sections before making your selection.

☒ Life sciences ☐ Behavioural & social sciences ☐ Ecological, evolutionary & environmental sciences

For a reference copy of the document with all sections, see nature.com/documents/nr-reporting-summary-flat.pdf

Life sciences study design

All studies must disclose on these points even when the disclosure is negative.

Sample size	Sample size were provided in the manuscript. The sample size were determined based on litterature and standard experimental design. We did not perform sample size calculation.
Data exclusions	No data excluded
Replication	Experiments were repeated for three times independently.
Randomization	No randomization required.
Blinding	All experiments were done and analyzed blind.

Reporting for specific materials, systems and methods

We require information from authors about some types of materials, experimental systems and methods used in many studies. Here, indicate whether each material, system or method listed is relevant to your study. If you are not sure if a list item applies to your research, read the appropriate section before selecting a response.

Materials & experimental systems

n/a	Involved in the study	
☐	☒	Antibodies
☒	☐	Eukaryotic cell lines
☒	☐	Palaeontology and archaeology
☐	☒	Animals and other organisms
☒	☐	Clinical data
☒	☐	Dual use research of concern

Methods

n/a	Involved in the study	
☒	☐	ChIP-seq
☒	☐	Flow cytometry
☒	☐	MRI-based neuroimaging

Antibodies

Antibodies used	Anti-type I collagen (Affinity, NO. AF7001), anti-α-SMA (Abcam, NO. ab32575), anti-E-cadherin (Sigma, NO. MABT26), anti-BMP-7 (Sigma, NO. MAB4350), anti-Desmin (Proteintech, NO. 16520-1-AP), anti-Vimentin (Proteintech, NO. 60330-1-Ig), anti-YAP (Proteintech, NO. 13584-1-AP), anti-TAZ (Proteintech, NO. 23306-1-AP), anti-p-YAP (Affinity, NO. AF3328), anti-p-TAZ (Affinity, NO. AF4315), anti-MST1 (Affinity, NO. DF8430), anti-p-MST1 (Affinity, NO. AF3688), anti-LATS1 (Affinity, NO. AF7669), anti-p-LATS1 (Affinity, NO. AF7169), anti-NF2 (Abcam, NO. ab109244) and anti-GAPDH (Abcam, NO. ab9485).

Validation	The validation of commercial antibodies used in this study were posted in manufacturer's website.

Animals and other research organisms

Policy information about studies involving animals; ARRIVE guidelines recommended for reporting animal research, and Sex and Gender in Research

Laboratory animals	24 male C57BL/6J mice (6-8 weeks of age).
Wild animals	N/A
Reporting on sex	Only male C57BL/6J mice were included in this study.
Field-collected samples	N/A
Ethics oversight	Wenzhou Medical University's Animal Care and Use Committee approved all experiments conducted on animals in the Experimental Animal Center.

Note that full information on the approval of the study protocol must also be provided in the manuscript.
